# A reproducible ddRAD-seq protocol reveals novel genomic association signatures for fruit-related traits in peach

**DOI:** 10.1186/s13007-025-01415-3

**Published:** 2025-07-22

**Authors:** Najla Ksouri, Gerardo Sánchez, Carolina Font i Forcada, Bruno Contreras-Moreira, Yolanda Gogorcena

**Affiliations:** 1 Group of Genomics of Fruit Trees and Grapevine, Department of Pomology, Estación Experimental de Aula Dei-Consejo Superior de Investigaciones Científicas, Avenida de Montañana 1005, 50059 Zaragoza, Spain; 2 Biotechnology Lab, Estación Experimental Agropecuaria (EEA) San Pedro, INTA, Ruta N°9 Km 170, B2930 San Pedro, Argentina; 3 Department of Pomology, Estación Experimental de Aula Dei-Consejo Superior de Investigaciones Científicas, Avenida de Montañana 1005, 50059 Zaragoza, Spain; 4 Laboratory of Computational and Structural Biology, Department of Genetics and Plant Production, Estación Experimental de Aula Dei-Consejo Superior de Investigaciones Científicas, Avenida de Montañana 1005, 50059 Zaragoza, Spain

**Keywords:** Lead SNP, Prime candidate genes, Haplotype blocks, Fruit-related traits, Linkage disequilibrium, *Prunus persica*

## Abstract

Improving peach cultivars with superior traits is a primary objective of breeding initiatives. In this study, we aimed to elucidate the genetic basis of key agronomic and fruit-related traits using a reproducible ddRAD-seq protocol applied to a discovery panel of 90 peach accessions. Our customized workflow (available at https://github.com/najlaksouri/GWAS-Workflow) integrates three variant callers and tests up to seven models to perform a robust genome-wide association analysis (GWAS). This approach produced 13,045 high-confidence SNPs and identified Blink as the most suitable model, effectively balancing false positive and negative associations. A total of 16 significant associations signals were unveiled for six highly heritable traits (H^2^ > 0.5), including harvest date, fruit weight, flesh firmness, contents of flavonoids, anthocyanins and sorbitol. By assessing the allelic effect of significant markers on phenotypic attributes, nine SNP alleles were deemed favorable. Notably, a promising marker (SNC_034014.1_7012470) demonstrated simultaneous association with harvest date and fruit firmness, displaying a positive allelic effect on both traits. We anticipate that this marker can serve as a good predictor of firmer varieties. Candidate causal genes were shortlisted when fulfilling the following criteria: (i) position within the linkage disequilibrium block, (ii) functional annotation and (iii) expression pattern. A comprehensive bibliographic review of previously reported QTLs mapping nearby the associated markers allowed us to benchmark the accuracy of our approach. Despite the moderate germplasm size, ddRAD-seq allowed us to produce an accurate representation of the peach genome, resulting in SNP markers suitable for empirical association studies. Together with candidate genes, they lay the foundation for further genetic dissection of peach key traits.

**Supplementary Information:**

The online version contains supplementary material available at 10.1186/s13007-025-01415-3.

## Background

Peach is one of the most economically valued fleshy fruits worldwide (FAO, http://faostat.fao.org). The advances in the peach industry largely rely on fruit quality improvement in response to the market and consumers’ demands. The term quality may include all agronomical aspects and chemical compounds such as fruit size, firmness, sugar and acid concentration, etc. Some of those characteristics are thought to be monogenic, controlled by a single gene (fruit shape, hairiness, flesh color) [1–3] while others are polygenic, such as sugar content, fruit firmness, antioxidant concentration [4].

Breeding for polygenic quantitative traits is far from being a straightforward task. Thus, insights on genetic drivers controlling these traits and their inheritance are required to bridge the phenotype-genotype gap [5]. The development of molecular markers linked to desirable traits would considerably speed up the selection of superior plant varieties through marker-assisted selection (MAS) [6]. GWA studies have also revolutionized the breeding process by detecting the genetic loci underlying trait variations at a relatively high resolution. This approach has been successfully applied in many breeding programs. For instance, reliable DNA-markers were developed for pollen sterility and red skin color in peach [6]. The power and prediction accuracy of GWAS critically depends on various considerations, including phenotypic data quality, experimental sample size, linkage disequilibrium (LD) between genetic variants and population structure. If not adjusted properly, these factors may lead to spurious associations as well as masking the true ones. Another key factor while performing GWAS is the density and chromosome distribution of markers/SNPs along the reference genome.

Genotyping methods generally fall into three categories; whole genome resequencing, reduced representation sequencing, and SNP arrays [7]. While whole genome resequencing yields the highest number of SNP calls with adequate sequencing depth, it can be cost-prohibitive for large genomes. As a cost-effective alternative, SNP arrays are widely employed, facilitating the detection of thousands of SNPs in a single assay [7]. In the case of peach, commercially available arrays IPSC peach 9 K [8] and IPSC peach 18 K [9] have been extensively used to explore the genetic diversity and to support the breeding process [3, 10]. Despite their usefulness, the major drawback of SNP genotyping arrays consists in their ascertainment bias [11]. In other words, they tend to limit the discovery of new variants beyond those identified in the reference discovery panel and used to construct the array. This bias can potentially lead to skewed interpretation of the genetic data. Furthermore, the design and optimization of efficient SNP probes requires a well-assembled reference genome, adding to the time-consuming nature of this process.

Driven by the rapid advancement in high-throughput technologies, reduced representation sequencing such as restriction-associated DNA (RAD) sequencing and its derivative double digest restriction-site associated DNA (ddRADseq) emerged to overcome both cost and ascertainment bias [12]. ddRADseq relies on the use of a pair of restriction enzymes to limit the sequencing effort to a subset of evenly distributed loci in the genome. Moreover, by selecting the optimal enzyme combination, repetitive DNA can be less targeted, thereby reducing the computational burden associated with aligning genomes with highly repetitive segments. Unlike other genotyping methods, prior genomic information is strictly not required for ddRADseq [12]. Nonetheless, as demonstrated in this study, its power is significantly enhanced when combined with a reference genome sequence. From a technical standpoint, a common challenge of ddRADseq is the high rate of missing calls, which can be effectively addressed through genotype imputation.

Here we share a reproducible analysis protocol that we tested with ddRAD-seq genotyping to accurately infer high-confidence SNPs within a discovery panel of 90 *Prunus persica* accessions. Subsequently, GWAS was carried out to elucidate genomic loci associated with 16 fruit traits. To optimize the analysis and to mitigate potential limitations arising from the germplasm size, we implemented several optimizations: (1) we deliberately selected geographically distant, unrelated peach accessions to maximize genetic variance within our panel; (2) SNPs were called using three independent variant callers (BCFtools, Freebayes and GATK) and only those consistently identified across all three approaches were retained for subsequent analysis, ensuring thus the robustness and reliability of our results; and (3) we rigorously evaluated multiple statistical models to effectively control for confounding factors and enhance the accuracy of genotype-to-phenotype associations.

While peach traits have been widely studied using various genotyping methods like SSRs [13], 9 K SNP array [3, 4, 14], 18 K SNP array [1, 10] and high-throughput resequencing technology [15]. To the best of our knowledge, this study presents the first reproducible and customizable protocol leveraging ddRADseq-derived SNPs for the characterization of their genetic architecture. We propose best practices for GWAS analysis, primarily focusing on a comparative approach for SNP calling and statistical model assessment. Furthermore, we demonstrate the utility of ddRAD-based genotyping in unveiling desirable alleles and genomic regions putatively responsible for trait variation. By contrasting our findings with those previously reported using the peach 9 K SNP array [14], we benchmark the accuracy of our approach.

## Methods

### Plant material and phenotypic evaluation

A total of 90 peach and nectarine accessions underwent double digest restriction-site associated sequencing (ddRAD-seq) followed by GWAS analysis. The germplasm panel comprises 73 landraces and 17 modern breeding lines originating from Spain, USA, France, Italy, New Zealand, and South Africa. All genotypes were grown under Mediterranean soil conditions at the Experimental Station of Aula Dei (CSIC) located at Zaragoza, Spain (41.7245° N, 0.8118° W) and evaluated during three fruiting seasons (2009–2011). Information about plant accessions is summarized in Additional file 1, Table S1.

The phenotypic data previously reported by Font i Forcada et al. [14] were re-analyzed in the present study. Briefly, 16 traits were evaluated by randomly harvesting 20 fruits from each cultivar at the commercial maturity for 3 years. Traits were split into two categories. Agronomic features included harvest date (HvD; Julian days), fruit weight (FW; grams), flesh firmness (FF; Newton), soluble solids content (SSC; ºBrix), titratable acidity (TA; grams malic acid/100 g flesh weight) and ripening index (RI; SSC/TA). Besides, biochemical variables comprised vitamin C (Vit C; mg of ascorbic acid/100 g flesh weight), total phenolics (Phen; mg of gallic acid equivalents/100 g flesh weight), contents of flavonoids (Flv; catechin equivalents/100 g flesh weight) and anthocyanins (ACNs; cyanidin-3-glucoside/kg flesh weight), sucrose (Suc; g/kg flesh weight), glucose (Glu; g/kg flesh weight), fructose (Fruc; g/kg flesh weight), sorbitol (SRB; g/kg flesh weight), and total sugars (TS; g/kg flesh weight) and relative antioxidant capacity (RAC; μg trolox equivalents/g flesh weight).

Broad sense heritability (H^2^) for each trait, defined as the ratio of total genetic variance to phenotypic variance, was estimated using variability R package v0.1.0 [16]. To assure real associations, traits with H^2^ > 0.5 were considered for subsequent analysis. Descriptive statistics and the analysis of variance (ANOVA) were performed using SPSS software v25 (Inc. Chicago, IL, USA). The distribution of phenotypic data was assessed using Shapiro–Wilk test and non-normal distributions were transformed using bestNormalize package (v1.8.3) [17]. Pairwise phenotypic correlations were computed annually, between years, and based on the mean values, using the cor () function of R.

### DNA extraction and enzyme evaluation

Genomic DNA was extracted from leaves using the DNeasy Plant Mini Kit (Qiagen, Dusseldorf, Germany) following the manufacturer’s recommendations. DNA concentration and quality were checked using PicoGreen®dye and measured in a fluorospectrometer. Whole-genome genotyping was carried out using ddRAD-seq by combining rare and frequent cutters to digest genomic DNA; *Pst1* and *Mbol* in this case, as described in peach by Aballay et al. [18]. This enzyme pair was selected based on a comparative evaluation of in vitro and in silico digestions using multiple rare/frequent enzyme pairs, where the first enzyme represents the rare cutter and the second the frequent one. The tested combinations included *SphI/MspI*, *SphI/MboI*, *EcoRI/MspI*, *EcoRI/MboI*, *PstI/MspI*, and *PstI*/*MboI*. These enzyme pairs were selected for their ability to generate a high number of loci within the 300–400 bp size range and importantly, for including at least one methylation-sensitive enzyme per pair to avoid sampling repetitive, heavily methylated regions. In silico digestion conducted using the simRAD R package and peach genome reference were consistent with the in vitro experiments. Both approaches demonstrated that *PstI*/*MboI* pair produced the highest number of fragments within the desired region, making it the most efficient choice for genome complexity reduction in peach [18, 19]. Only fragments with sticky ends from both enzymes were retained for library construction.

### ddRAD libraries preparation and sequencing

DNA libraries were constructed at the Genomic Unit at IABiMo INTA-CONICET (Argentina) using a ddRAD-seq protocol optimized for peach. Briefly, genomic DNA was digested using *Pst1/Mbol* restriction enzyme pair. The resulting fragments were ligated to 24 barcoded adapters each designed with sticky-end modifications complementary to the overhangs generated by the respective restriction enzymes. The ligated DNA fragments were pooled in equal quantity into eight pools of 24 samples. To ensure accurate fragment sizing, an automatic size selection was performed using a 2% agarose cassette on the SAGE ELF (Sage Science, Inc., Beverly, MA, USA). Fragments averaging 450 bp were collected from one well, and an additional purification step with 0.8 × Ampure XP beads was employed to remove any fragments smaller than 300 bp. Libraries were PCR-amplified using indexed primers containing Illumina-compatible sequences and an 8 bp index to tag each pool. This protocol was adapted from Aguirre et al. [19]. Finally, paired-end sequencing (2 × 250 bp) was carried out on an Illumina NovaSeq 6000 at CIMMYT, Mexico. The raw reads are available at the European Nucleotide Archive (ENA) under BioProject accession PRJEB62784.

### Data processing and alignment

Raw reads were de-multiplexed and trimmed using the “process-radtag” module from STACKS suite (v2.59) [20]. Redundant reads, commonly referred to as PCR duplicates were removed prior to the alignment using “clone_filter” tool from the same suite. This step is critical not only for improving data accuracy but also for reducing the computational burden during the genome alignment. The resulting de-duplicated reads were then mapped to *Prunus persica* reference genome v2 (GCF_000346465.2, retrieved from NCBI RefSeq [21] using BWA-mem (v0.7.17) [22]. Specifically, the -M and -R parameters were applied to ensure compatibility with GATK and to enhance the alignment quality. The -M option marked shorter split alignments as secondary and the -R added the read group tags to each read, for proper sample identification during variant calling. Aligned reads were sorted and indexed using SAMtools [23], to be ready for downstream variant calling.

### Variant discovery pipeline

Variant calling was conducted in a single-sample mode testing the performance of three variant callers: BCFtools (v1.7) [23], Freebayes (v1.0.0) [24] and GATK-HaplotypeCaller (v4.2.3.0) [25]. Raw SNPs were subjected to standard quality filtering using bcftools filter to reduce false-positive calls. Variants were retained only if they met all of the following criteria: a mapping quality (MQ > 40), indicating that the reads were confidently and uniquely aligned to the reference genome; a variant quality score (QUAL > 30), reflecting high confidence in the accuracy of the SNP call, and a minimum depth of read (DP ≥ 5), ensuring that each SNP was supported by at least 5 sequencing reads. Consequently, clean SNPs from each calling method were merged by position and by reference/alternative alleles into multi-sample VCF files. SNPs resulting from the intersection of multi-samples VCFs were considered as highly accurate calls and were inspected to remove multi-allelic variants and those assigned to scaffolds. Then, they were filtered by call rate > 80% and residual missing genotypes were imputed with beagle’s default settings (v4.1) [26]. The imputation accuracy was evaluated in Tassel (v5.0) [27] by masking 1% of the genotype and calculating the error rate. SNPs with (MAF > 0.05) were selected as a final call set to determine the population structure and marker-trait associations. SNP identifiers were created by concatenating their assigned chromosome and their base pair position (eg: SNC_034014.1_7012470).

### Linkage disequilibrium and population structure

Intra-chromosomal LD was calculated using Plink (v1.9) [28] as a measure of *Pearson* correlation coefficient (r^2^) between marker-pairs. For each chromosome, the LD decay was determined by plotting the r^2^ values against the physical distance (Mbp) and the trend line was fitted using the average of r^2^ variation across 1 Mbp bins. The LD decay extent was estimated as the physical distance at which the average r^2^ dropped to half its maximum value at the 90th percentile (r^2^max, 90 = 0.5) [29]. Thus, the r^2^ threshold was set at (r^2^_(1/2)_ max, 90 = 0.25). Subsequently, SNPs showing strong LD were pruned in Plink by delimiting a window of 10 SNPs, removing one of the SNPs pair with r^2^ > 0.25 and then shifting the window 5 SNPs forward repeatedly. Genetic distance and kinship matrix between pairs of genotypes were computed using the centered identity-by-state method implemented in Tassel [27].

LD-pruned SNPs were selected to infer the population stratification of the GWAS panel using two complementary approaches. First, the Bayesian clustering algorithm implemented in fastSTRUCTURE (v1.04) [30] was tested on predefined K subgroups ranging from 1 to 10. The optimal K value was estimated based on the lowest cross validation error. Then, principal component analysis was computed with SmartPCA (v1.1.0) R-package [31].

### Genome wide association study

For association mapping, seven statistical models, ranging from single to multi-locus, were simultaneously tested in GAPIT (v3.1.0) [32]. The single locus models included general linear model (GLM), mixed linear model (MLM), compressed MLM (CMLM), and settlement of MLMs under progressively exclusive relationship (SUPER). Multi-locus algorithms comprised multiple loci mixed linear model (MLMM), fixed and random model circulating probability unification (FarmCPU), and Bayesian-information and linkage-disequilibrium iteratively nested keyway (Blink). Except for GLM, where no genotype relatedness was considered, the top four PCs components and kinship were introduced as covariates to control false positives. The statistical model, best fitting the data, was chosen based on the quantile–quantile (Q-Q) plot and the number of significant markers. Significantly associated markers were shortlisted based on the Bonferroni correction (− log_10_(0.05)/13045 = 5.42) and Manhattan plots were generated accordingly using CMplot package (v4.2.0) [33]. Statistically significant SNPs explaining at least 10% of the phenotypic variance (%PVE) were considered as most promising predictions and used for LD block analysis. Moreover, markers with the largest PVE contributions are hereinafter referred to as ‘lead SNPs’.

### Annotation of SNP effects and identification of favorable alleles

First, genomic coordinates of SNPs were used to query Ensembl Plants REST services to obtain annotations of their effect on nearby genomic features. We used the Ensembl Variant Effect Predictor (VEP) [34] and a modified version of recipe R8 [35]. Next, allelic effect of significant SNP loci on trait variation was estimated through pairwise comparisons between the phenotypic values of the different genotypes: homozygous reference (0/0), heterozygous (0/1) and homozygous alternative (1/1). An allele was considered as “favorable” when a significant increase of the phenotypic value was observed between the homozygous reference and the remaining genotypes. Pairwise comparisons were run using the Games-Howell test and *P*-values were adjusted for multiple testing using the FDR method. The results were visualized using the ggstatsplot R-package (v0.9.1) [36].

### LD-block analysis and identification of candidate genes

Significant SNPs were examined to identify potential candidate genes. Initially, emphasis was placed on identifying whether polymorphisms were localized in genic regions. Thereby, SNPs were mapped based on their physical positions to the *Prunus persica* genome (GCF_000346465.2). SNPs anchoring genes were labeled as ‘prime candidates’.

Strong candidate genes were shortlisted using a three-steps filtering approach: (i) falling within the LD-block region harboring the significant SNPs, (ii) being functionally related to the trait of interest and (iii) being differentially expressed in fruit tissue. Expression data was retrieved from a recent study by Ksouri et al. [37] which defined modules of co-expressed genes across different peach tissues and under various experimental conditions. Differentially expressed genes were those outlined in fruit experiments, particularly under cold storage and chilling injury.

LD-blocks were identified within 250 Kbp windows at either side of the lead sites. Block boundaries were delimited using a solid spine partitioning approach from the LDBlockShow tool [38]. A block is defined as a group of SNPs that are in strong LD (D’ ≥ 0.7) with the first and last marker of the same block. A value of D’ = 0 denotes complete linkage equilibrium, suggesting a frequent pairwise recombination between markers. Conversely, a value of D’ = 1 indicates a complete LD. Note that D’ and r^2^ are common measures of non-random association between two or more loci; while D’ refers to the co-inheritance of two alleles, r^2^ considers the allele frequency to distinguish between common and rare. Identified LD blocks were therefore scanned for candidate genes via NCBI genome data viewer [39].

### QTLs review for fruit quality traits in peach

To benchmark the accuracy of our results, an exhaustive bibliographic review of previously reported QTLs mapped in the same linkage group as the associated markers was done. In a practical term, if an associated SNP is located nearby or within a QTL interval, then it’s considered as highly accurate and likely to segregate with the observed trait. In cases where precise QTL boundaries in base pairs were unavailable, we employed either the nearest marker or the co-localizing marker as a reference. Herein, the nearest marker is defined as the closest one with a maximum of 5 cM from the QTL hotspot while the co-localizing marker resides within the QTL boundaries. Moreover, we calculated the physical distance separating our predicted associated markers from the reported QTLs. Finally, we compared these distances with those obtained from a previous study using the same phenotypic data and peach material, albeit genotyped using the 9 K SNP array [14]. This comparative analysis provided valuable insights into the consistency and reliability of our results, contributing to the advancement of our understanding of the genetic architecture underlying complex traits in peach.

## Results

### Phenotypic analysis and heritability

**Fig. 1:**
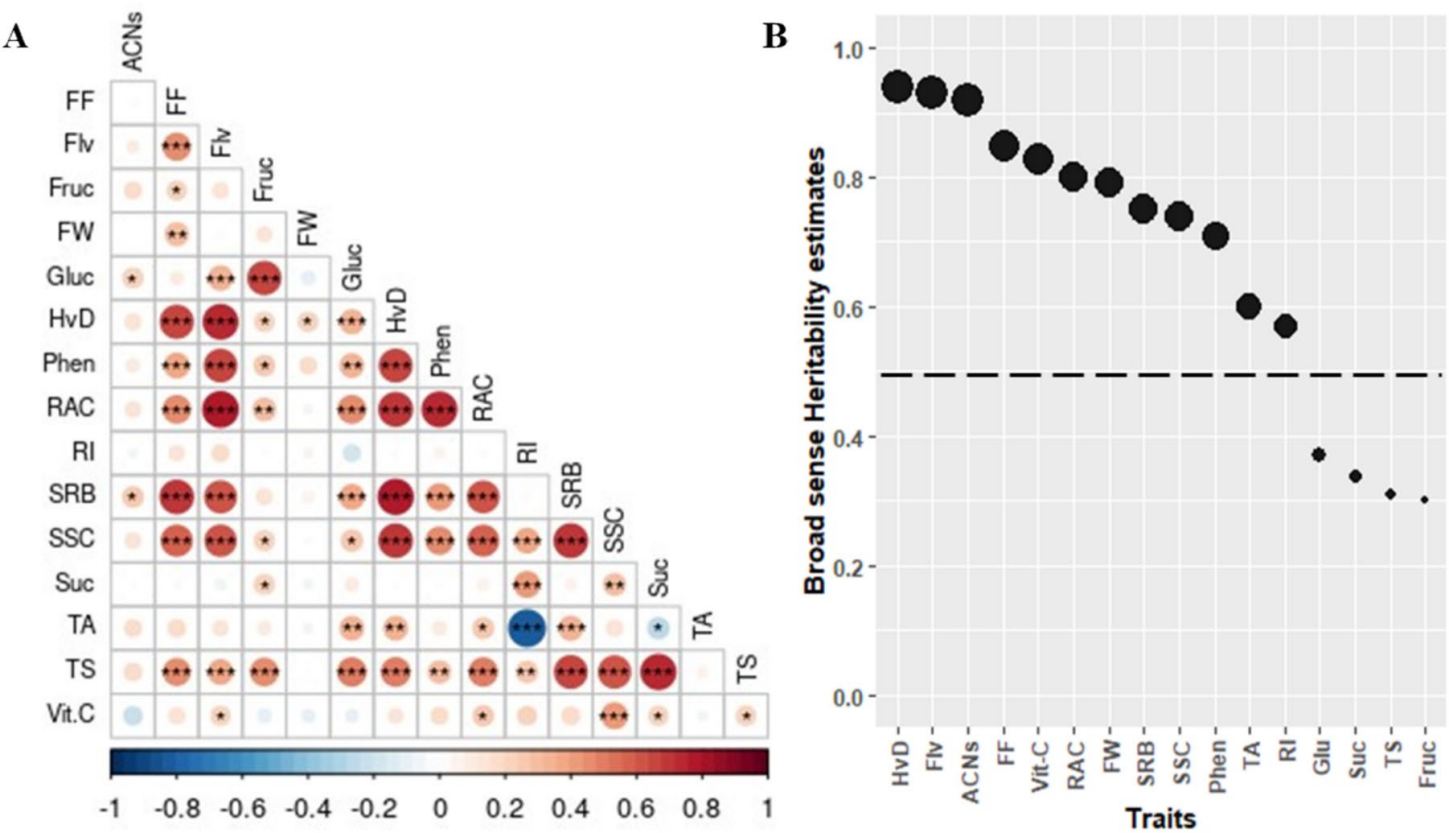
**A** Phenotypic correlation estimates across mean phenotypic data. Significant positive and negative correlations are displayed in red and blue respectively at three different levels (*P < 0.05, **P < 0.01 and ***P < 0.001). **B** Broad sense heritability estimates over 3 years. Dashed horizontal line corresponds to heritability threshold (H^2^ = 0.5). Abbreviations are as follows: harvest date (HvD), fruit weight (FW), flesh firmness (FF), soluble solids content (SSC), titratable acidity (TA), ripening index (RI), content of vitamin C (Vit C), total phenolics (Phen), anthocyanins (ACNs), flavonoids (Flv), sucrose (Suc), glucose (Glu), fructose (Fruc), sorbitol (SRB) total sugars (TS) and relative antioxidant capacity (RAC)

Phenotypic data collected for the GWAS panel in 3-year experiments are summarized in (Additional file 1, Table S2). Broad sense heritability was estimated and categorized as weak (H^2^ < 0.5) and strong (H^2^ > 0.5). Our results reflected high heritability for most of the traits (Fig. 1A), indicating that their phenotypic variability among the individuals was mainly driven by the genetic components. However, contents of glucose, fructose, sucrose and total sugars (TS) were found to be less heritable and were therefore left out of the analysis. For the remaining traits, pairwise *Pearson* correlations within-years, between-years and using the mean values appeared to be steady in terms of sign and magnitude (Additional file 2, Figure S1). Notably, harvest date (HvD) exhibited strong positive correlations with flesh firmness, sugar contents measured as (SSC, and sorbitol) and antioxidant activity measured as (RAC, flavonoids and phenols). As expected, significant positive interaction was also reported between the HvD and fruit weight. Moreover, a strong positive correlation was also observed between total phenolics and flavonoids. Indeed, flavonoids are the largest group of naturally occurring phenolic compounds in plants. Both compounds showed a significant positive interaction with (RAC) suggesting that they could be used as a good indicator of antioxidant properties in peaches. Analysis of variance components was conducted to detect the effect of genotypes and years. While genotypes exhibited significant effects for all traits, the year effect was irrelevant for HvD, FF, Phen, Flv, and ACNs (Additional file 1, Table S2). Considering the consistent phenotypic correlations among years, GWAS we conducted using the mean values for all traits passing the heritability cutoff. Source code, documentation and detailed results can be accessed at https://github.com/najlaksouri/GWAS-Workflow.

### SNP genotyping

To construct an informative SNP panel, polymorphic sites were called in individual sample mode using three different algorithms. This comparative approach was chosen to address the existing variability in SNP detection associated with reduced-representation sequencing methods such as the ddRAD-seq. Raw calls were subjected to standardized quality thresholds to mitigate the effect of sequencing and/or alignment flaws. Post-filtered calls from each pipeline were converted to multi-sample VCFs. We observed notable differences in variant yield across tools. GATK-HaplotypeCaller (HC) outperformed both Freebayes and BCFTools in terms of computational time and sensitivity yielding a total of 233,535 SNP calls (see repository https://github.com/najlaksouri/GWAS-Workflow). Freebayes ranked second, generating 166,080 SNPs, followed by BCFTools with 148,998 SNPs. To improve reliability, we retained only the intersection of SNPs detected by all three callers, resulting in 56,430 high-confidence variants (~ 32%). Subsequently, multi-allelic and scaffold variants were excluded, and additional filters based on missing call rate and minor allele frequency (MAF) were applied (Table 1). A final set of 13,045 high-quality SNPs was retained for subsequent analysis.

**Table 1:** SNPs count and filtering steps

Applied filters	Retained SNPs
Clean multi-samples SNPs from GATK-HaplotypeCaller	233,535
Clean multi-samples SNPs from Freebayes	166,080
Clean multi-samples SNPs from BCFtools	148,998
Intersected set	56,850
Removing scaffold SNPs	56,647
Removing multi-allelic sites	56,430
Missing call rate < 20%	26,188
Minor Allele Frecuency > 0.05	13,045

To gain insight into the genomic distribution and functional impact of the selected SNPs, we performed annotation using the Variant Effect Predictor (VEP) tool. The majority of the SNPs were located in non-coding regions: 21% upstream, 9% downstream, 26% intronic and 8% intergenic. A smaller proportion of SNPs were tagged as 3ʹ and 5ʹ untranslated (UTR) variants (Additional file 2, Figure S2). Within the coding region, 11% were classified as synonymous and 13% as missense mutations suggesting a potential alteration in protein sequence. This comprehensive annotation provides valuable insights into the functional consequences of genetic variation across the peach genome.

### SNP distribution and LD decay

**Fig. 2:**
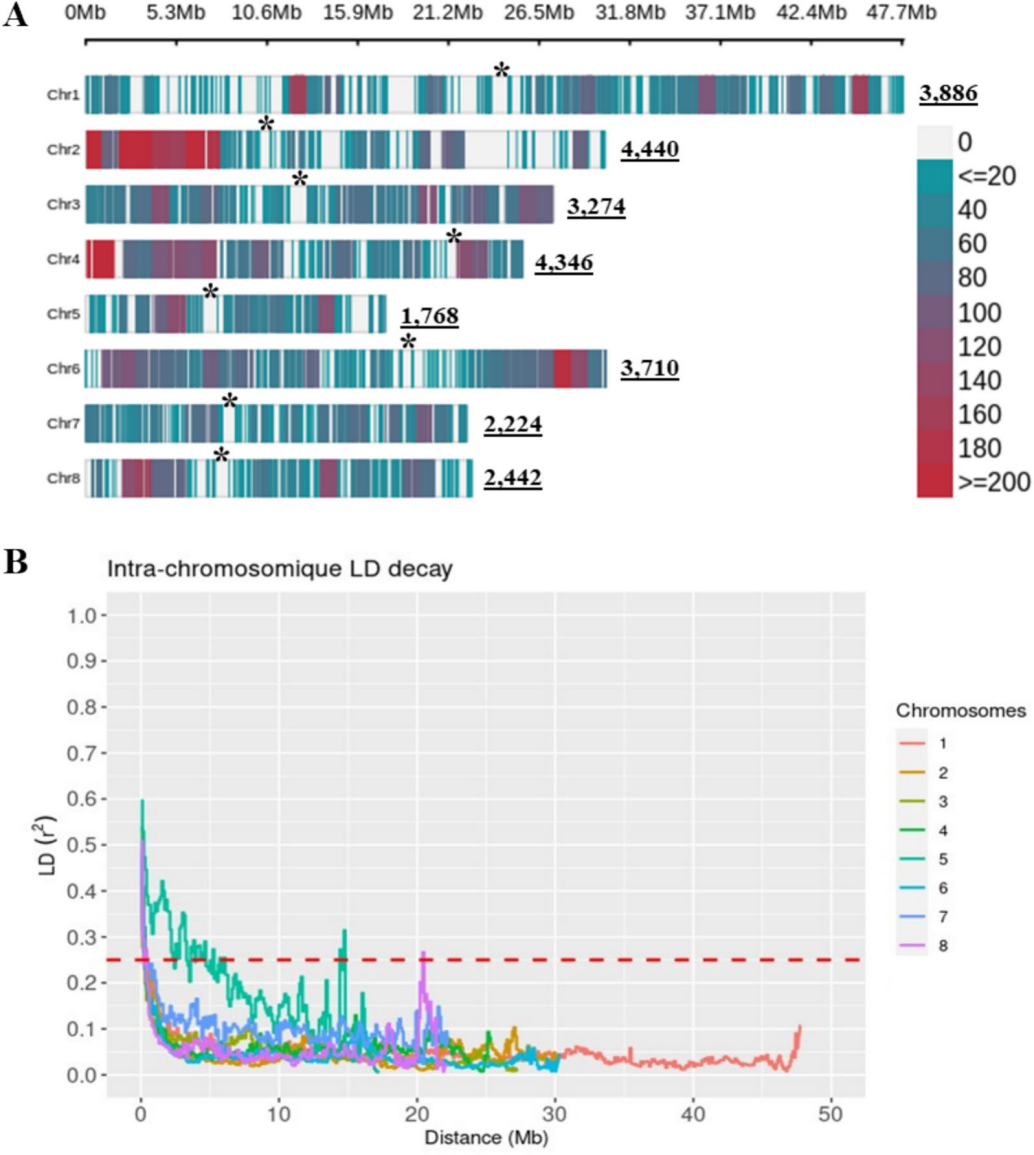
SNPs density plot and intra-chromosomique linkage disequilibrium decay. **A** SNPs density across the eight peach chromosomes. The horizontal axis shows the chromosome length in (Mbp) and the different colors reveal the SNP density per window of 1 Mb. Underlined numbers correspond to the total number of markers per chromosome (Chr). The asterisks highlight the putative position of centromeres predicted as follows: Chr 1 = (~ 21 Mbp), Chr 2 = (~ 8 Mbp), Chr 3 = (~ 12 Mbp), Chr 4 = (~ 24 Mbp), Chr 5 = (~ 7 Mbp), Chr 6 = 1: (~ 15 Mbp), Chr 7 = (~ 7 Mbp) and Chr 8 = (~ 10 Mbp). **B** chromosome-wide LD decay of r^2^ (y-axis) over the physical distance in Mbp (x-axis). Each colored line represents a smoothed r^2^ for all marker pairs on each chromosome. The horizontal dashed red line indicates a cut-off r^2^ = 0.25

The distribution of polymorphic sites was calculated within adjacent windows of 1 Mbp and provided a genome-wide coverage estimate along the eight peach chromosomes. As illustrated in Fig. 2A, markers were unevenly partitioned throughout the genome with the highest number of mapped SNPs on chromosome 2 (4440), whereas chromosome 5 displayed the lowest count (1768). Notably, SNPs were preferentially concentrated within the short arms of chromosomes 2 and 4. In contrast, conspicuous gaps were observed along the long arm of chromosome 2 and several regions on chromosome 1. Gaps highlighted with asterisks correspond to predicted centromeric regions, consistent with prior findings by Verde et al. [40].

Linkage disequilibrium analysis conducted at chromosome level inferred a fast decay in function of the increased physical distance (Fig. 2B and Additional file 2, Figure S3). However, this pattern of rapid decline was notably absent in case of chromosome 5. Despite being the shortest chromosome with the lowest number of SNPs, the LD decay rate on chromosome 5 persisted over a longer distance (~ 4.7 Mbp) compared to other chromosomes, where LD decayed within a range of approximately 200–500 Kbp. Initially, we hypothesized that this distinctive LD decay pattern could be attributed to an uneven distribution of SNP density. To test this hypothesis, we randomly selected 20 SNPs in each 2 Mbp window, yet the slow LD decay persisted as depicted in (Additional file 2, Figure S3). We thus cannot rule out that there are less recombination events between loci along chromosome 5 (chr 5).

### Population structure

**Fig. 3:**
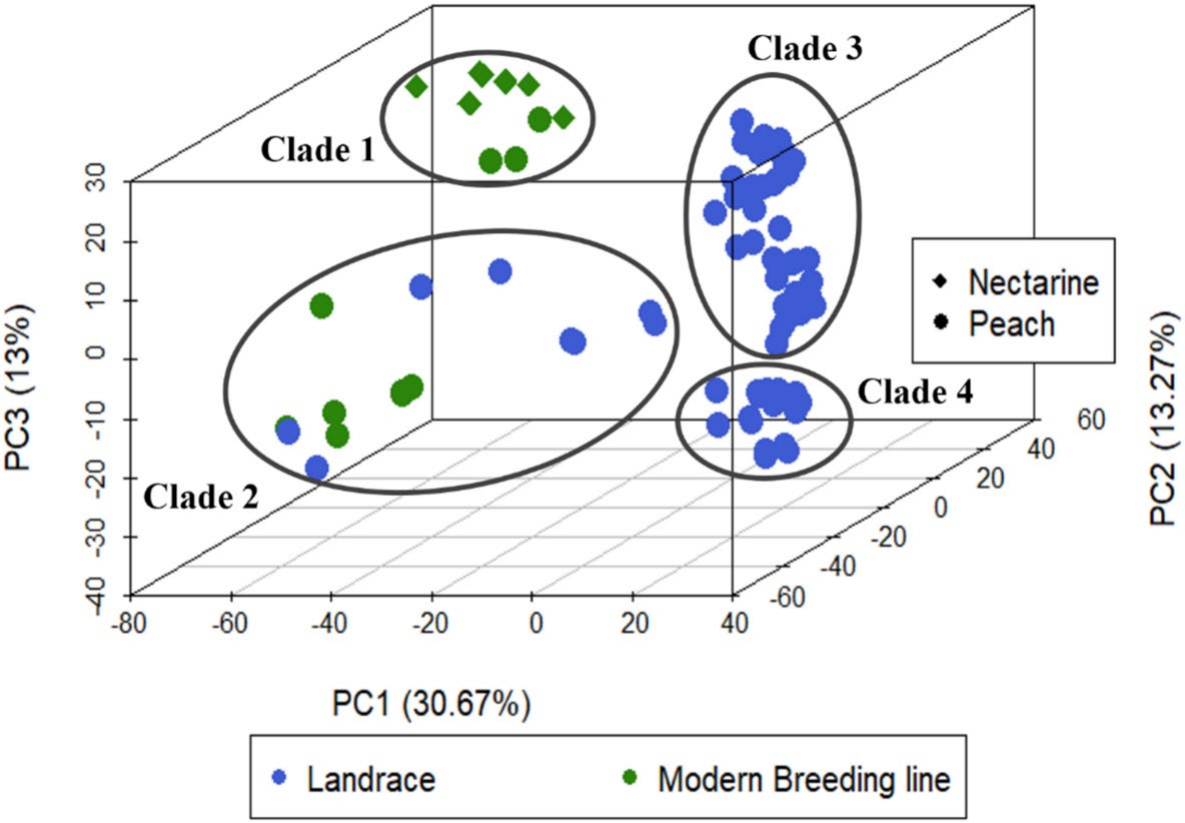
Principal component analysis (PCA) of 90 *Prunus persica* accessions. Blue and green colors indicate respectively landraces and modern breeding lines. Shapes correspond to peach and nectarine genotypes. Clades 1 to 4 correspond to the inferred sub-populations (K = 4)

PCA analysis separated the germplasm panel into 4 sub-populations based on the genetic origin (landrace vs modern breeding line) and fruit type (peach vs nectarine) (Fig. 3). The top four principal components (PCs) collectively explained 60% of the total genetic variation. Clade 1 on the top left corner, grouped exclusively modern breeding lines of peach and nectarine. Notably, the geographical origin of most accessions within this clade was traced back to North America as detailed in (Additional file 1, Table S1).

Clade 2 represents a diverse genetic entity gathering both landrace and bred peach varieties. Genotypes within clade 2 were originated from Spain and North America suggesting a higher level of genetic admixture, likely arising from the exchange of the germplasm materials. In contrast, clades 3 and 4 were consisted of landrace peach accessions primarily originated from different regions of Spain, Europe and South Africa. Additionally, a neighbor joining (NJ) tree also identified four clear clusters, as illustrated in (Additional file 2, Figure S4). Comparable results were obtained from fastSTRUCTURE and are further detailed in the GitHub repository https://github.com/najlaksouri/GWAS-Workflow.

### Critical evaluation of GWAS models

Genome wide association studies may be susceptible to bias in the presence of measurement errors. False positive and negative associations arising from population structure or/and family relatedness may lead to erroneous conclusions. The examination of Q–Q plots can be used as a straight visual inspection to determine the appropriate statistical method controlling the confounding effects. In fact, Q–Q plots illustrate the distribution of markers under the null hypothesis, by plotting the observed − log_10_ *P-*values (y-axis) versus the expected − log_10_ *P-*values (x-axis). If a sharp diagonal line is observed, then the null hypothesis is respected, and no significant associations are reported. However, an upper deviated tail from the diagonal line would likely indicate true associations. Upward inflation close to the line’s origin indicates suspicious false positives while downward deflated tail suggests false negatives.

**Fig. 4:**
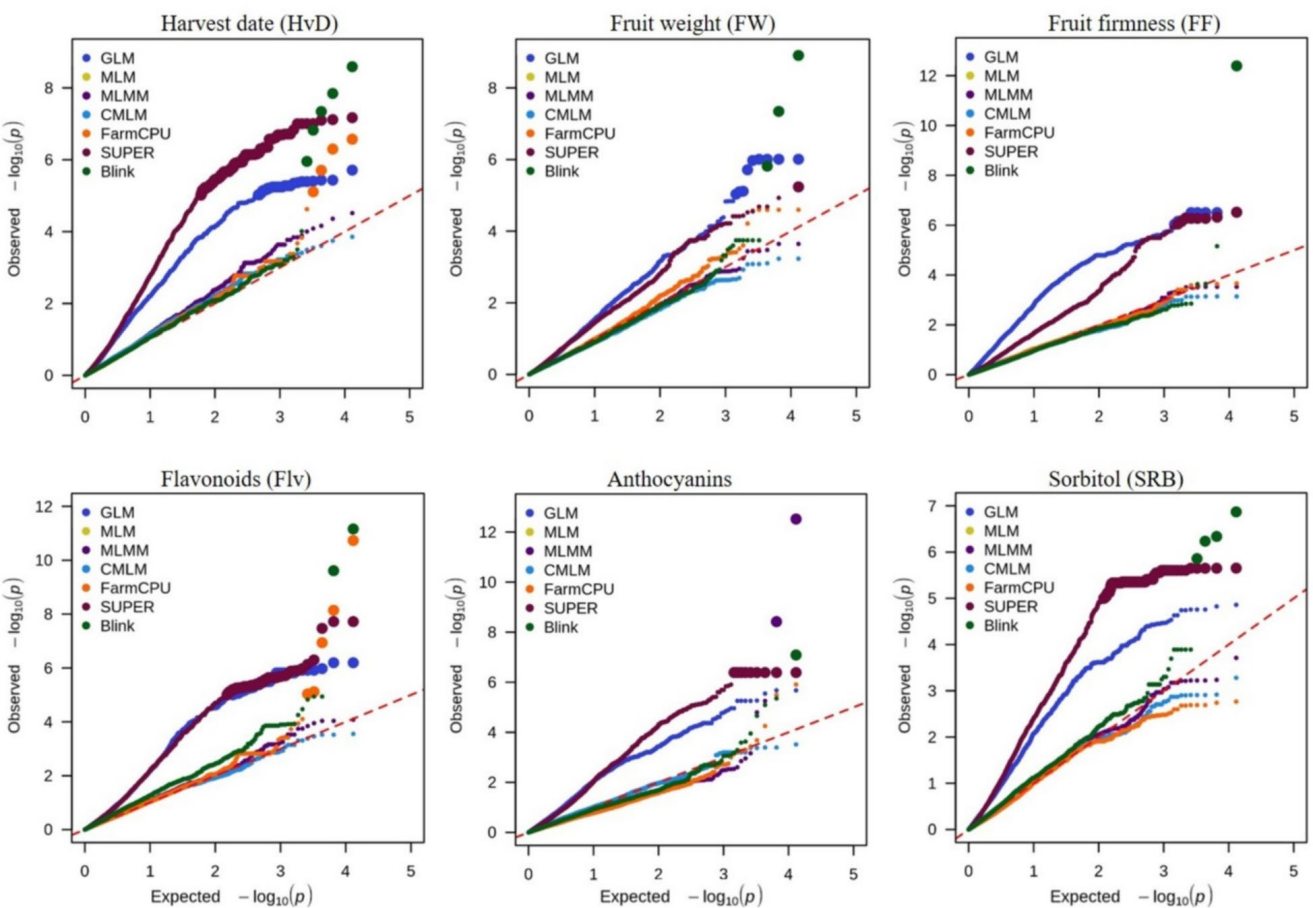
Q-Q plot comparison between the GWAS models implemented in GAPIT: General Linear Model (GLM), Mixed Linear Model (MLM), Compressed MLM (CMLM), Settlement of MLM under Progressively Exclusive Relationship (SUPER), Multiple Loci Mixed Linear Model (MLMM), Fixed and random model Circulating Probability Unification (FarmCPU) and Bayesian-information and Linkage-disequilibrium Iteratively nested keyway (BLINK). Note that MLM and CMLM models are overlaid. For each SNP, the expected -log_10_ transformed *P*-value (x-axis) is plotted against the observed -log_10_ transformed *P*-value (y-axis). The red dashed diagonal line corresponds to the expected Q-Q trendline under the null hypothesis (no association with the phenotype). Larger size dots refer to SNPs statistically associated with a trait. For clarity, only phenotypic traits with significant associations were represented

We empirically evaluated the adjustment of seven models to our data and in Fig. 4, we plot their Q–Q behavior for significantly associated traits. Despite yielding statistically significant associations, represented as bigger dots, both single locus models GLM and SUPER exhibited prominent inflation beyond the expected null line. This deviation starting close to the origin indicates false positive predictions due to confounding effects (population stratification or genotype relatedness). MLM and CMLM multi-locus models showed matching *P*-value distributions, therefore their Q–Q plots were overlaid. Except for harvest date, where the null hypothesis cannot be rejected with neither inflated nor deflated *P*-values, MLM and CMLM unveiled downshifted line tails when assessed with the rest of traits. Such a result may indicate that these tests were able to reduce false positive associations, but likely yielded false negative ones. Another complex model (MLMM) was found to follow the null hypothesis with both harvest date and flavonoids; nonetheless a slightly downward tail was discerned for fruit weight and sorbitol content. Although FarmCPU proved to be the best-fitting model for identifying marker-trait associations with harvest date and flavonoids, it lacked the same statistical power for other traits. Conversely, the observed *P*-values generated by Blink (green color) closely aligned with the diagonal line with clearly deviated tails toward the y-axis for all six aforementioned traits. Overall, Blink emerged as the best calibrated model, effectively controlling false positive and false negative effects. Therefore, we consider Blink as the most suitable model, best adjusted with our phenotypic data. Henceforth, the GWAS results are based on it.

#### Harvest date (HvD)

**Fig. 5:**
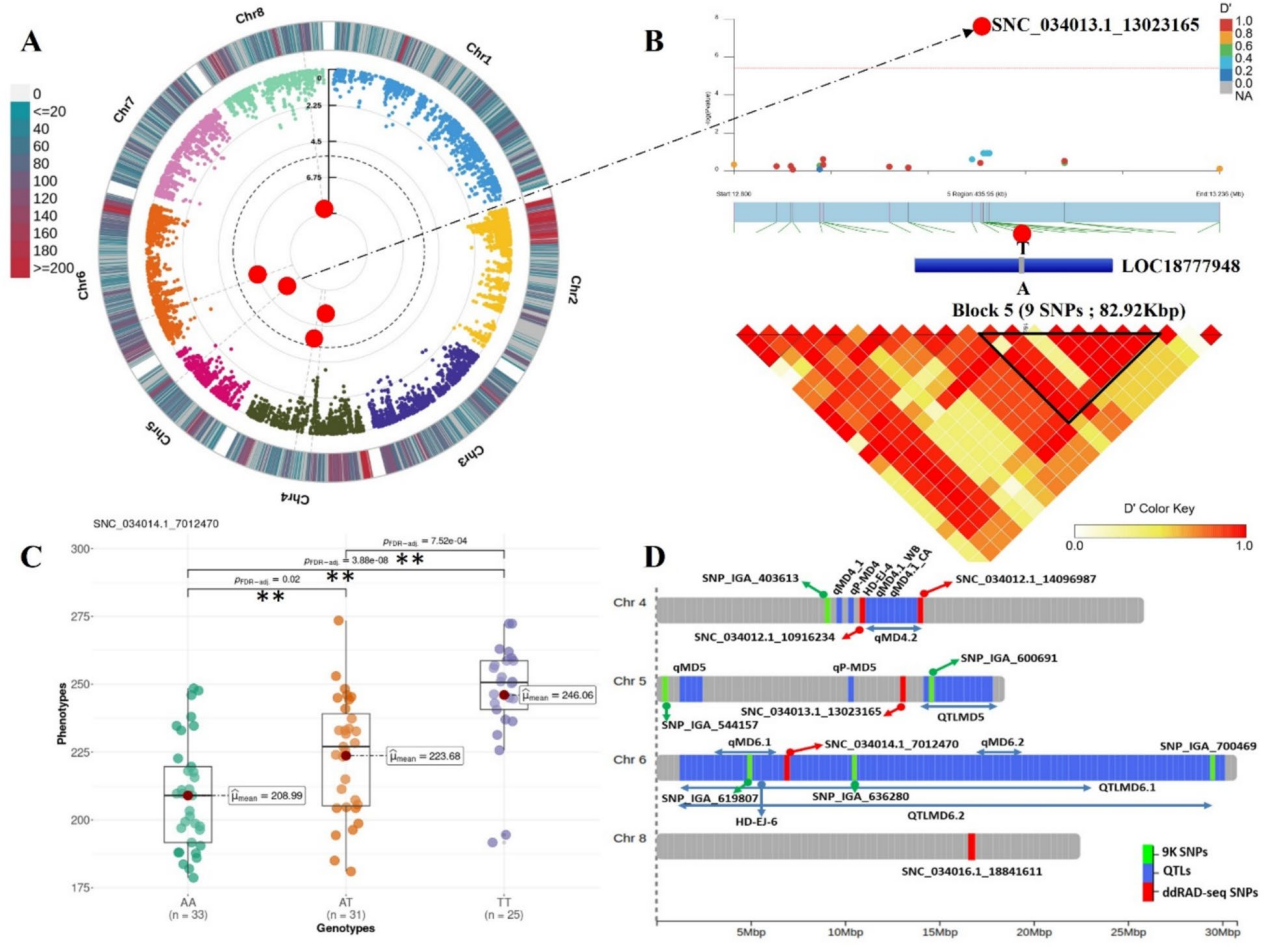
Genome Wide Association and LD block analysis for harvest date (HvD). **A** Circular Manhattan plot and association signals based on Blink model. Black dashed circular line corresponds to the Bonferroni adjusted threshold (− log_10_(*P*) = 5.42). Red and large size dots correspond to statistically associated SNPs. Degradation from blue to red indicates the SNP density per 1 Mbp window across peach chromosomes. **B** Locus-specific Manhattan plot (upper panel) and LD heatmap (bottom panel) within 250 Kbp on either side of the lead SNP (SNC_034013.1_13023165). The prime candidate gene is represented as a blue box which in this case contains a single coding exon, where blue fragment refers to the exon. Pairwise LD measurements are displayed as D’ values with a color transition from yellow to red. **C** Boxplot depicting allelic effect of significant SNP on trait variation. Herein we highlight the SNP commonly affected Harvest date and fruit firmness. Mean value for each genotype is indicated by red circle and ** indicates significant pairwise comparison calculated by Games Howel test (*P* ≤ 0.05). **D** Genomic distribution of significant ddRAD-derived SNPs (red), reviewed QTLs in the literature (blue) and 9 K array derived SNPs (green)

The GWAS analysis resulted in five SNPs meeting the Bonferroni-adjusted threshold (Fig. 5). Two SNPs were located on chr 4 and tagged as (SNC_034012.1_10916234, G/T) and (SNC_034012.1_14096987, A/C). Their allelic effect is summarized in (Additional file 2, Figure S5), with the former associated with delayed harvest and the later with early one. Another significant marker was found on chr 5 (SNC_034013.1_13023165, T/A). Although covering the highest portion of %PVE (Table 2), no significant allelic effect was observed. This lead SNP was mapped within the first exon of the gene *LOC18777948*, annotated as encoding a germin-like protein according to the NCBI RefSeq curated gene models. However, based on the annotation submitted by the Peach Genome Functional Genomics (PGF), this genomic region corresponds to *Prupe.5G138500*, for which the lead SNP is positioned in the upstream region. This discrepancy highlights a common challenge in plant genomics, where gene models may differ slightly between annotation databases due to differences in transcript models or genome annotations. Another significant site was identified on chr 6 and was labeled as (SNC_034014.1_7012470, A/T). The allelic effect on phenotypic variation highlighted that both heterozygous and homozygous genotypes carrying the alternate allele (T) were lately harvested with respectively 14 and 37-days of delay (Fig. 5C). In contrast, the intergenic SNP located on chr 8 (SNC_034016.1_18841611, A/G), showed approximately 39-days acceleration in harvest date for heterozygous accessions (Additional file 2, Figure S5).

**Table 2:** Information on significantly associated SNP markers with fruit-related traits in *Prunus persica*

Traits	SNP identifier	Alleles	Chr	Position	%PVE	SNP location [effect]
**HvD**	SNC_034012.1_10916234	G/T	4	10,916,234	10.7	Intergenic
	SNC_034012.1_14096987	A/C	4	14,096,987	24.5	Intronic
	**SNC_034013.1_13023165**	T/A	5	13,023,165	30.0	Exonic
	SNC_034014.1_7012470	A/T	6	7,012,470	2.8	Intergenic
	SNC_034016.1_18841611	A/G	8	18,841,611	10.2	Intergenic
**FW**	SNC_034011.1_26371177	T/A	3	26,371,177	16.9	Exonic
	**SNC_034014.1_1805059**	A/G	6	1,805,059	22.0	Intergenic
	SNC_034016.1_16407694	A/C	8	16,407,694	18.7	5ʹUTR
**FF**	**SNC_034014.1_7012470**	A/T	6	7,012,470	33.9	Intergenic
**Flvs**	**SNC_034010.1_643430**	T/C	2	643,430	35.7	Intergenic
	SNC_034014.1_3066620	G/T	6	3,066,620	14.5	Exonic [missense]
**ACNs**	**SNC_034013.1_12838635**	G/T	5	12,838,635	52.9	Exonic [missense]
**SRB**	SNC_034009.1_27061825	T/C	1	27,061,825	9.0	Exonic [missense]
	SNC_034010.1_3682553	G/C	2	3,682,553	11.8	Intronic
	SNC_034014.1_28343678	G/A	6	28,343,678	10.4	Intronic
	**SNC_034016.1_18841643**	G/A	8	18,841,643	14.0	Intergenic

Alleles are shown on the forward strand as reference/alternate. Please note that all SNP locations are based on the NCBI Refseq annotations

Variant in bold refers to ‘lead SNP’, explaining the highest proportion of phenotypic variance (PVE). Chromosome (Chr), Harvest date (HvD), fruit weight (FW), flesh firmness (FF), and contents of flavonoids (Flvs), anthocyanins (ACNs) and sorbitol (SRB)

LD block analysis of associated markers revealed several candidate genes (Additional file 1, Table S3), including cell wall modification (*Prupe*.*8G197700*: galacturonosyltransferase and *Prupe*.*8G199700*: cell division control protein), cytochrome P450 enzymes (*Prupe*.*8G196800*, *Prupe*.*8G196900*, *Prupe*.*8G197100* and *Prupe*.*8G197300*), UV-photoreceptor (*Prupe*.*4G185200*) and ethylene-responsive transcription factor (*Prupe*.*8G198700*).

#### Fruit weight (FW)

**Fig. 6:**
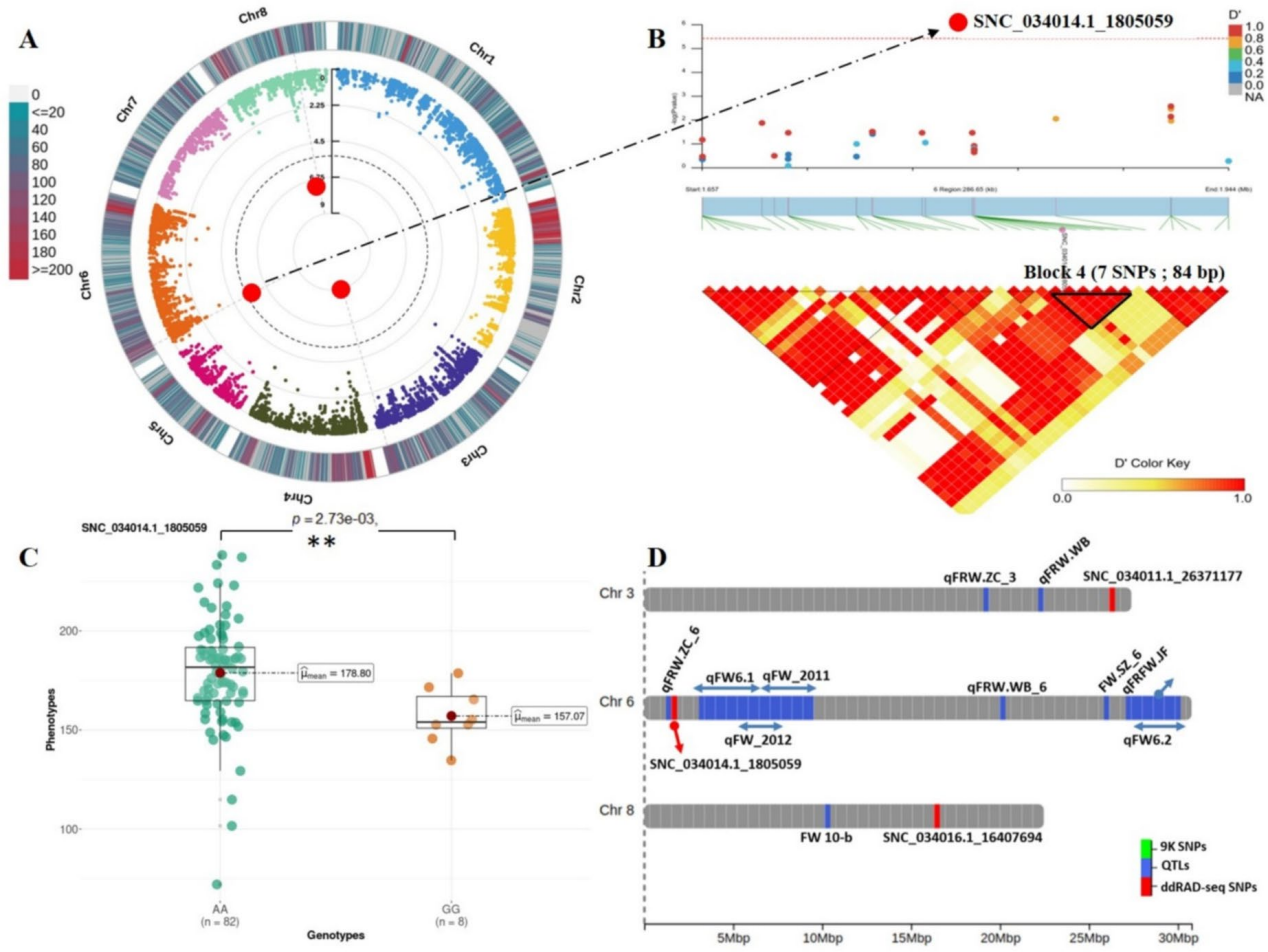
Genome Wide Association and LD block analysis for fruit weight (FW). **A** Circular Manhattan plot and association signals based on Blink model. Black dashed circular line corresponds to the Bonferroni adjusted threshold (− log_10_(*P*) = 5.42). Red and large size dots correspond to statistically associated SNPs. Degradation from blue to red indicates the SNP density per 1 Mbp window across peach chromosomes. **B** Locus-specific Manhattan plot (upper panel) and LD heatmap (bottom panel) within 250 Kbp on either side of the lead SNP. Pairwise LD measurements are displayed as D’ values with a color transition from yellow to red. **C** Boxplot depicting allelic effect of lead SNP on trait variation. Mean value for each genotype is indicated by red circle and ** indicates significant pairwise comparisons calculated by Games Howel test (*P* ≤ 0.05). **D** Genomic distribution of significant ddRAD-derived SNPs (red) and reviewed QTLs in the literature (blue)

Significant marker-trait associations were detected on three chromosomes: chr 3 (SNC_034011.1_26371177, T/A), chr 6 (SNC_034014.1_1805059, A/G) and chr 8 (SNC_034016.1_16407694, A/C). The explained variance oscillated between 17 and 22%, with SNC_034014.1_1805059 identified as the lead intergenic marker (Table 2). The allelic effect of this lead marker (A/G) was found to be unfavorable, with the allele G associated with weight loss (~ 22 g) in homozygous accessions (Fig. 6C). A similar negative effect was observed with the SNP on chr 3 (T/A). This effect was evident in both heterozygous and homozygous genotypes carrying the alternate allele, leading to a reduction of 24 and 53 g in fruit weight, respectively. Only one 5ʹUTR marker mapped on chr 8 (A/C) was found to have a positive effect in homozygous alternate accessions (Additional file 2, Figure S6). According to the LD block results, the lead SNP fell within the fourth block, a small interval (84 bp) overlapping no genes (Fig. 6B). Nonetheless, the associated SNPs overlapped protein-coding genes. Among them, genes encoding β-galactosidase (*Prupe*.*3G298200*), α-galactosyltransferase (*Prupe*.*3G298800*), thymidylate kinase (*Prupe*.*3G301400*) and transcription factors (GTE8: *Prupe*.*3G301300* and trihelix GT-4: *Prupe*.*3G300500*) (Additional file 1, Table S3).

#### Flesh firmness (FF)

**Fig. 7:**
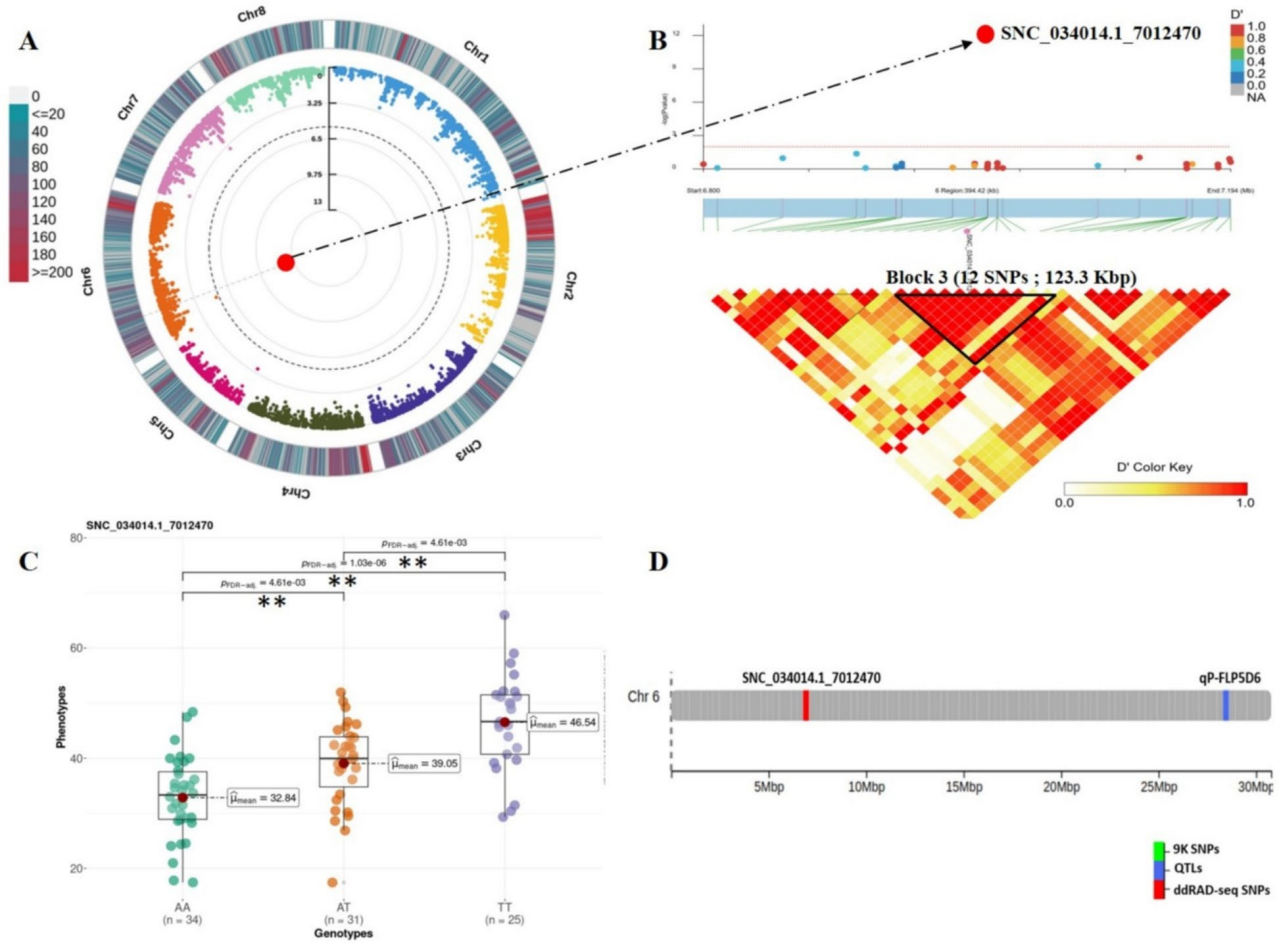
Genome Wide Association and LD block analysis for flesh firmness (FF). **A** Circular Manhattan plot and association signals based on Blink model. Black dashed circular line corresponds to the Bonferroni adjusted threshold (− log_10_(*P*) = 5.42). Red and large size dots correspond to statistically associated SNPs. Degradation from blue to red indicates the SNP density per 1 Mbp window across peach chromosomes. **B** Locus-specific Manhattan plot (upper panel) and LD heatmap (bottom panel) within 250 Kbp on either side of the lead SNP. Pairwise LD measurements are displayed as D’ values with a color transition from yellow to red. **C** Boxplot depicting allelic effect of lead SNP on trait variation. Mean value for each genotype is indicated by red circle and ** indicates significant pairwise comparisons calculated by Games Howel test (*P* ≤ 0.05). **D** Genomic distribution of significant ddRAD-derived SNPs (red) and reviewed QTLs in the literature (blue)

A single intergenic marker (SNC_034014.1_7012470; A/T) detected on chr 6 was statistically linked to flesh firmness and explained 33.9% of the total phenotypic variance (Table 2, Fig. 7A). This polymorphism resulted in a significant increase in fruit firmness in both heterozygous and alternate homozygous genotypes, underscoring the favorable effect of the alternate allele (T) on fruit firmness (Fig. 7C). Notably, this SNP was the only one simultaneously associated with both harvest date and firmness and thereby potentially explaining the high correlation observed between these traits (Fig. 1B). Peach accessions harboring the allele (T) either in homozygous or heterozygous states, were classified as late-harvested and firm peach accessions. By examining 250 Kbp upstream and downstream the lead marker, it was found to reside in block 3, making it a relevant region to seek for candidate firmness-related genes (Fig. 7B). Based on their functional annotation, six genes were selected as potential candidates, including *Prupe.6G100500* encoding an E3 ubiquitin-protein ligase, *Prupe.6G101100* corresponding to vegetative cell wall protein, *Prupe*.*6G101600* annotated as aquaporin PIP2 and *Prupe.6G102300* encoding homeobox-leucine zipper transcription factor (Additional file 1, Table S3).

#### Flavonoids (Flvs)

The Manhattan plot displayed two peaks statistically associated with flavonoids content (Additional file 2, Figure S7A). The first peak, (SNC_034010.1_643430, T/C), was identified in the intergenic region of chr 2 and displayed a favorable effect of the alternate allele (C). Indeed, for both heterozygous (TC) and homozygous alternate (CC) genotypes approximately a two-fold increase in the flavonoid content was observed (Additional file 2, Figure S7C). The second associated SNP (SNC_034014.1_3066620; G/T) was located on chr 6 and physically mapped on the first exon of *Prupe.6G041500*; a candidate gene encoding a non-specific lipid-transfer protein-like (Additional file 1, Table S3). The average flavonoids content in alternative homozygous peach accessions (TT) was significantly enhanced compared to the reference homozygous individuals (GG) (Additional file 2, Figure S8). Thus, the T allele can be considered as a favorable one. Based on LD block results, we annotated a total of 14 genes (Additional file 1, Table S3). According to their biological function and tissue-specific expression, we narrowed the list to a few promising ones, including two genes encoding transcription factors (*Prupe*.*2G009100*, bHLH and *Prupe*.*6G041400*, bZIP).

#### Anthocyanins (ACNs)

GWAS analysis for anthocyanin content revealed a single significant peak on chr 5 exceeding the threshold (Additional file 2, Figure S9A). This locus tagged as (SNC_034013.1_12838635; G/T) resides within exon 2 of *Prupe*.*5G134900*, a candidate gene encoding a B3 domain-containing transcription factor. Consequently, *Prupe*.*5G134900* was considered a prime candidate gene. This marker explained a substantial portion of the variation (53%) but exhibited an unfavorable effect on anthocyanin content (Additional file 2, Figure S9C). Specifically, pairwise comparisons of allelic effects revealed significantly lower anthocyanin content in homozygous alternate individuals (TT) compared to the reference genotype (GG). Screening for genes residing within LD block resulted in three further candidate genes involved in different biological functions (*Prupe.5G134200*, *Prupe*.*5G134800* and *Prupe.5G135200*) (Additional file 1, Table S3).

#### Sorbitol (SRB)

Four significant association signals dispersed on different chromosomes were predicted to affect the sorbitol content (Table 2 and Additional file 2, Figure S10). On chr 1, a missense SNP (SNC_034009.1_27061825; T/C), explained the lowest proportion of phenotypic variation. The SNP on chr 2 (SNC_034010.1_3682553; G/C), in the third intron of a gene encoding a flowering time control protein (*Prupe*.*2G0303400*), explained 12% of the PVE.

Similarly, (SNC_034014.1_28343678; G/A) was located on chr 6 and mapped on the intronic region of *Prupe.6G320000*, a gene encoding a serine/arginine rich factor. Both *Prupe*.*2G0303400* and *Prupe*.*6G320000* are suggested as plausible sorbitol-related genes. The lead SNP explaining the highest PVE (14%) was identified in an intergenic region of chr 8 (SNC_034016.1_18841643; G/A).

Except for (SNC_034014.1_28343678) the remaining loci were observed to have desirable effect on sorbitol content (Additional file 2, Figure S11). We identified 26 genes distributed in 250 Kbp on either side of each associated SNP. Among them, some were discovered to be over-expressed in the fruit (Log_2_FC > ǀ3ǀ), including genes encoding heavy metal-associated isoprenylated proteins (*Prupe*.*2G033600*, *Prupe*.*2G033700* and *Prupe.6G321400*), pectinesterases (*Prupe*.*6G318500*), exonucleases (*Prupe*.*6G316100*), dormancy-associated proteins (*Prupe.6G319600*), cell cycle checkpoint control proteins (*Prupe*.*6G321300*) and the E3 ubiquitin-protein ligase RNF4 (*Prupe*.*8G199600*). A cluster of four cytochrome P450 encoding genes was also identified. This plethora of genes may shed light on several key processes that are subject to influence the sorbitol biosynthesis.

## Discussion

### Performance of variant callers

SNP discovery in plant genomes holds significant importance for various applications such as marker-assisted selection (MAS), genomic selection, and phylogenetic analysis. In our study, we implemented a comprehensive SNP discovery pipeline, leveraging ddRAD-seq reads and employing three alternative variant callers: BCFtools, Freebayes, and GATK-HaplotypeCaller. These tools were applied to previously mapped paired-end reads. SNP calling is inherently error prone, with problems arising from various sources such as sample processing (library preparation, PCR amplification), sequencing and computational analysis [41]. To mitigate the risk of false positive variants, stringent filtering criteria were applied based on mapping and call quality, read depth, call rate, and minor allele frequency (MAF). Though either calling tool can be adapted, we observed variability in the number of high-quality SNPs identified by each tool. Notably, GATK-HC exhibited the highest sensitivity in SNP calling, followed by Freebayes then BCFtools. This outperformance is likely due to GATK-HC’s reliance on local *de-novo* assembly of haplotypes in active regions [25]. In other terms and unlike the rest of tools, whenever GATK encounters regions with substantial evidence of variation relative to the reference, it discards the existing mapping information and reassembles the read mappings. Our results are consistent with previous findings in *Arabidopsis thaliana*, where GATK-HC demonstrated superior accuracy compared to BCFtools [42]. Additionally, according to Ksouri et al. [43], GATK-HC showed the lowest proportion of false positives compared to both Freebayes and BCFtools. The underlying algorithms likely contribute to these variations, as GATK-HC and Freebayes are Bayesian variant detectors, while BCFtools mpileup utilizes Hidden Markov Models. Although having an extensive format requirement (e.g.: read group specification in the input header), GATK-HC seems to be more precise dealing with ddRAD-seq mapped reads in peach. To ensure the highest confidence in our results, we deliberately opted for a conservative approach retaining only SNPs identified by all three tools and thus prioritizing specificity over sensitivity. While this approach increases confidence in SNP accuracy, it may also lead to the loss of informative variants potentially limiting the power of ddRAD-seq. This trade-off was considered necessary in the context of our relatively small germplasm panel (90 individuals), where minimizing false positives was a key priority.

### Comparison between GAPIT models

Choosing a statistically reliable model is another fundamental step for a successful GWAS. Population structure and genetic relatedness are confounding factors that can inflate the rate of ambiguous associations and reduce the statistical power. When ignored, they lead to substantial inflation of *P*-values as highlighted in the GLM model (Fig. 4). Despite including PCA components and kinship as covariates, SUPER model had also a large number of false positives. This may be explained by the fact that both GLM and SUPER are single-locus approaches failing to catch true associations when dissecting complex traits. Similar results were observed in *Arabidopsis thaliana*, when testing the flowering time with the naïve model (GLM) [44]. In contrast, MLM-based methods, including MLM, CMLM, and MLMM were found to adjust for false positives at the cost of failing to find any significant marker (Fig. 4).

Based on the inspection of the Q–Q plot, both FarmCPU and Blink emerged as well-fitted models, effectively addressing overfitting and minimizing the false positives. While FarmCPU detected significant associations with only two traits (HvD and Flv), Blink consistently uncovered associations across all six traits (HvD, FW, FF, Flv, ACNs, and SRB). Remarkably, in our dataset, Blink demonstrated superior performance in terms of the number of associations and the significance level. These results align well with those reported by the GAPIT team, confirming Blink’s statistical superiority over FarmCPU [45, 46]. However, Blink's effectiveness has primarily been demonstrated on species such as *Arabidopsis thaliana*, human, maize, mouse, and pig, with *Arabidopsis* being the only dicotyledonous species (as peach), albeit with a smaller genome size [47]. As the performance of Blink might be influenced by several factors, including species, sample size, genetic diversity, and trait complexity, its application to peach and ddRAD-seq with our protocol is novel and will likely guide other researchers in the community.

### Comparison of genotyping strategies: SNP arrays vs. reduced representation sequencing

Genotyping methods differ in cost, genomic resolution, number of SNPs detected, and required technical and bioinformatics resources. Choosing the most appropriate strategy requires balancing these trade-offs. Among the most widely used approaches in plant genomics and in *Prunus* species in particular, are SNP arrays and Reduced Representation Sequencing (RRS) methods. RRS targets only a fraction of the genome, making it a cost-effective alternative to whole-genome resequencing. Popular RRS methods include Genotyping-by-Sequencing (GBS) and double-digest RAD-seq (ddRAD-seq), both of which are widely used in GWAS studies due to their flexibility and moderate cost [7]. While GBS and ddRAD-seq share the principle of restriction enzyme digestion and de novo SNP discovery, ddRAD-seq offers finer control over genome complexity reduction. It uses two enzymes and a defined size selection step, enhancing reproducibility and reducing unwanted genomic regions. In contrast, standard GBS typically uses a single enzyme (e.g., *ApeKI*) and omits size selection leading to uneven coverage and a higher proportion of missing data. This missingness requires more hands-on time and greater bioinformatics expertise particularly for alignment, variant calling, and filtering. This complexity further increases in polyploid species, where sequencing depth must be significantly higher (e.g., 4–6× for diploids, 50–100× for polyploids such as strawberry or sugarcane) [48]. This difference in protocol complexity is also reflected in cost. In our ddRAD-seq genotyping experiment, we spent approximately $70 per sample (15 × sequencing depth), excluding DNA extraction and downstream analysis). GBS typically ranges between $35–60 per sample. The higher cost of ddRAD-seq is primarily due to its more labor-intensive library preparation, which involves dual digestion, adapter ligation, size selection, and additional quality control steps. These wet-lab steps require approximately 2–3 full working days and involve greater reagent consumption. However, this increased effort is justified by the notable advantages that ddRAD-seq offer. Notably, it provides greater scalability and customization as different enzyme combinations can be used to target specific regions and/or avoid repetitive or methylated DNA, especially when including at least one methylation-sensitive restriction enzyme. This represents a key improvement over GBS, which tends to oversample repetitive elements due to its simpler and less selective protocol.

On the other hand, SNP arrays, provide a high-throughput and cost-effective genotyping platform with relatively low per-sample costs (typically $20–60, including hybridization and scanning but excluding DNA extraction). For example, genotyping costs can be approximately $41 per almond sample (in 384-sample format) or $35 per cherry sample (in 96-sample format). The lab workflow is highly streamlined and can be completed in a single working day making them well-suited for large-scale and routine genotyping efforts.

From a bioinformatics standpoint, SNP array analysis is simple and efficient. Genotype calls are usually provided by the platform (e.g., Affymetrix Axiom or Illumina Infinium) significantly reducing computational effort. Alternatively, users can process the output themselves upon receiving the cell intensity files [48]. Following the respective best practices workflow, the process is straightforward and well-documented. Based on our personal experience, even for a first-time user, the analysis can be completed within 1–3 h. However, SNP arrays come with important limitations. Most notably, they are restricted to predefined variants, meaning they only capture polymorphisms known at the time of array design. This introduces ascertainment bias, potentially overlooking novel or rare variants, especially in genetically diverse or uncharacterized populations (e.g. wild relatives) [7, 11]. In the *Prunus* research community, another important limitation of SNP arrays is their restricted species coverage and declining commercial availability. Indeed, arrays exist only for three species: almond, cherry, and peach. These include the 6 K and combined 6 K + 9 K SNP arrays for cherry, the 70 K array for almond, and the 9 K and 18 K arrays for peach. However, several of these genotyping tools such as the 6 K and 6 K + 9 K cherry arrays, and the 9 K peach array are no longer commercially available. This issue underlines the reliance of array-based genotyping on commercial availability and industrial support. In contrast, sequencing-based approaches such as GBS and ddRAD-seq remain sustainable over time, as long as the laboratory protocols and analysis pipelines are well-documented. Finally, the total cost of genotyping depends on several factors beyond the chosen method, including sample number (bulk pricing), service provider (academic or commercial), and additional services such as DNA extraction, quality control, or data formatting.

### Marker-trait association for the target traits

Out of 16 phenotyped traits, we made a deliberate decision to focus our efforts on traits that are more likely to be influenced by genetic factors rather than environmental ones. Therefore, we selected 12 traits with high heritability for genetic association analysis. This approach allowed us to prioritize traits with a stronger genetic basis, increasing the likelihood of detecting significant genetic associations while minimizing the potential impact of environmental noise. Using Blink, 16 significant loci were inferred and distributed as follows: harvest date (chr 4, 5, 6 and 8), fruit weight (chr 3, 6 and 8), flesh firmness (chr 6), flavonoids (chr 2 and 6), anthocyanins (chr 5), and sorbitol (chr 1, 2, 6, and 8). Promising candidate genes were selected when residing within the LD block containing the significant loci, known to be related to the targeted trait and being over-expressed in fruit tissue. Our results were further discussed in comparison with Font i Forcada et al. [14] which studied the same phenotypic data and germplasm material but genotyped using the 9 K SNP array instead and less comprehensible bioinformatic analysis. As both studies were performed on a panel of 90 accessions, our results will benefit for confirmation analyses on larger germplasm sets.

#### Harvest date

Peaches and nectarine are generally harvested at physiological maturity, then ripening off the trees. Harvest date and maturity date are frequently used as synonyms and are expressed in Julian days. HvD specifically refers to the day when a certain percentage of peaches reach maturity, while MD represents the time interval from the first day of the calendar year until the harvest date [49]. In our study, we identified five association signals for HvD, with two located on chromosome 4 and the remaining signals distributed across chromosomes 5, 6, and 8.

As established by Eduardo et al. and Dirlewanger et al. [50, 51] major QTLs controlling maturity date have been reported on linkage groups LG4 and LG6 (Additional file 1, Table S4). Particularly, a major QTL on LG4 referred to as qMD4.1 showed a pleiotropic effect on fruit weight and firmness [50, 52]. Interestingly, our marker SNC_034012.1_10916234 mapped at (~ 10.91 Mbp), was overlapping the (qMD4.1_CA) locus from C × A progeny spanning the interval between 10.87–12.09 Mbp [52]. This same QTL from W × By progeny (qMD4.1_WB) was found 65 Kbp from our marker (Fig. 5D). Interestingly, a recent study by Da Silva Linge et al. [53] identified and validated a predictive KASP marker for a major QTL (Ppe.RPT/SSC‑1) associated with ripening time and soluble solids concentration on chr 4 (10.98–11.30 Mbp). This region overlaps with our qMD4.1 signals, further reinforcing the importance of chromosome 4 in peach ripening and maturity control. In the same vein, SNC_034012.1_10916234 was delimited by one downstream (HD-EJ-4) [54] and two upstream quantitative loci (qP-MD4) [55] and (qMD4_1) [56] mapped respectively at 0.5, 5.3 and 1,245 Kbp from the SNP’s coordinate (Fig. 5D). Likewise, the second marker on chr 4 (SNC_034012.1_14096987) mapped at (~ 14.09 Mbp) was found within the genomic region of strong confidence QTL (qMD4_2) spanning the interval (11.20–14.10 Mbp) [57]. Contrasting with associated SNPs from the 9 K assay [14] our markers appear to be more confident as they are located within the QTL boundaries which supports their reliability. Altogether, we anticipate that the aforementioned SNPs on chr 4 could be integrated as promising markers for HvD breeding goals. As well, we conclude that LG4 seems to be a chromosomal hotspot hosting a cluster of major QTLs associated with the maturity date. QTLs influencing maturity date were also detected on LG4 in stone fruits, for instance, sweet cherry [58]. Therefore, we believe that this trait could be controlled by orthologous loci within *Prunus* species.

Marker ‘SNC_034013.1_13023165’ mapped on chr 5 (~ 13.02 Mbp) was supported by an adjacent locus (QTLMD5) spanning the region (14.38–17.64 Mbp) [59] and other distant signals (qP-MD5 and qMD5) [55, 57]. Significant markers from 9 K array [14] were found to be physically closer to the QTLs (Fig. 5D and Additional file 1, Table S4). Finally, the significant SNP on chr 6 ‘SNC_034014.1_7012470’ was residing within two QTL intervals QTLMD6.1 and QTLMD6.2, supporting it [59]. Similar findings were observed with 9 K-associated markers.

Multiple candidate genes potentially influencing the harvest date were shortlisted (Additional file 1, Table S3). Most importantly, an ethylene-responsive transcription factor (*Prupe.8G198700*). Ethylene-responsive elements are relevant in climacteric fruits and have been proposed as candidate genes for fruit maturation date in different *Prunus* species [54, 60]. We also identified a cell wall remodeling gene encoding galacturonosyltransferase. This finding is in consonance with [60] defining a galacturonosyltransferase as a candidate gene for late harvested cultivars.

#### Fruit weight

Fruit weight is a quantitative trait with great importance in peach breeding. Previous investigations into peach genetics have unveiled the multifaceted nature of FW, controlled by multiple quantitative trait loci (QTLs) dispersed across all chromosomes [57, 60–63]. Leveraging GWAS, we pinpointed a significant SNP on chr 3 (~ 26.37 Mbp) positioned downstream of two QTLs, qFRW.ZC_3 and qFRW.WB, at distances of 4.07 Mbp and 7.27 Mbp, respectively (Fig. 6, Table S4). On chr 6, another significant marker was predicted at (~ 1.80 Mbp). This marker was flanked by two reliable QTLs (qFRW.ZC_6) [61] and (qFW6.1) [57] situated respectively at 387 bp and 1358 bp. Similarly, on chromosome 8, the marker SNC_034016.1_16407694, located at ~ 16.40 Mbp, was positioned downstream of a marker flanking QTL (FW 10-b) [63]. Our findings diverge from those of Font i Forcada et al. [14], where no associated loci were reported for FW on any chromosome (Additional file 1, Table S4). These results underscore the significance of our study in unraveling the genetic underpinnings of complex fruit traits and highlight the efficacy of ddRAD-seq genotyping in uncovering novel association signals.

Candidate genes prediction revealed two transcription factors, trihelix GT-4 (*Prupe.3G300500*) and GTE-8 (*Prupe.3G301300*). Transcriptional regulators are abundant in plant genomes and are implicated in various biological processes. Interestingly, trihelix genes are known to be photo-responsive proteins [64]. Considering the documented influence of light exposure on fruit size, shape, and quality [65], we hypothesize that trihelix TFs may play a role in regulating fruit weight in peaches. Moreover, cell wall enzymes such as β-galactosidase and α-galactosyltransferase may act as key components of cell wall turnover during stone fruit growth [66]. Finally, the strong upregulation of thymidylate kinase suggests a potential role in peach fruit development, as validated in rice, barley, and maize [67].

#### Flesh firmness

Firmness is a key textural indicator of peach quality and directly influences their shelf life. In our study, we identified a single firmness related locus SNC_034014.1_7012470 on chr 6. In the same LG6, at approximately 21 Mbp downstream of our marker, a firmness loss QTL (qP-FL5d6) was described (Fig. 7D and Additional file 1, Table S4). Additionally, another stable QTL (qP-FF6.1 m) was also detected over 2 years in related species, particularly in sweet cherry [58]. On the contrary, no significant association signals were found using the 9 K inferred SNPs and MLM model as indicated in Font i Forcada et al. [14].

Four genes were shortlisted as strong candidates encoding: ubiquitin-protein ligase (*Prupe.6G100500),* vegetative cell protein (*Prupe.6G101100*), aquaporin PIP2 (*Prupe.6G101600*) homeobox-leucine zipper protein (*Prupe.6G102300*). E3 ligase genes were found to be differentially expressed in either melting flesh or stony hard fruit during the ripening [68]. Aquaporins are transmembrane water transporters and water uptake within fruit is highly related with fruit firmness [69]. Thus, aquaporins could play a key role in maintaining cell turgor in peach fruit. Finally, homeobox-leucine zipper proteins were denoted as potential biomarkers for the ripening process in peach [70].

#### Flavonoid and anthocyanin contents

Flavonoids are major polyphenol compounds playing a central role in fruit color and flavor. Our analysis yielded two potential association signatures in chromosomes 2 and 6. These results go along with [15] highlighting that most lead SNPs linked with many flavonoid metabolites in peach were located on chr 2. Herein, SNC_034010.1_643430 was supported by two QTLs [63] identified in Venus × Bigtop progeny and named as ‘FLV 10-a’ and ‘FLV 10-b’ (Additional file 1 and 2, Table S4 and Figure S7D). It is well documented that flavonoid biosynthesis is a complex pathway, transcriptionally regulated by members of Myb and bHLH families [46]. Although no Myb encoding gene was found in our analysis, a highly up-regulated bHLH-TF was inferred and may be considered as a promising candidate gene involved in flavonoid regulation.

Anthocyanins, vital plant pigments within the flavonoid family, contribute significantly to the distinctive coloration of peach fruit and flesh [46]. While Myb10 transcription factor on LG3 is known to regulate anthocyanin biosynthesis, several anthocyanin-related QTLs have been identified on LG4, LG5, and LG6 [57, 61, 63]. In our analysis, a single lead marker on chromosome 5, ‘SNC_034013.1_12838635’, accounted for approximately 53% of the PVE (Table 2). This marker may serve as a preferred target for effective marker-assisted selection, as it is flanked by QTLs (qANT) [63], (qATCYN.ZC) [61], and (qPSC5) [57] (Additional file 2, Figure S9D). When genotyped with the 9 K array [14], no associated markers were detected on LG5.

Remarkably, our polymorphic marker was physically falling in the exonic region of *Prupe.5G134900*, a gene encoding a B3 domain-containing transcription factor. Although further validation is needed to ascertain the functional significance of this gene, we hypothesize that genetic control of anthocyanins may be influenced by B3 DNA-binding proteins. Notably, a B3 family transcription factor (*Prupe.6G041000*) was also identified as a candidate gene for flavonoid regulation in our analysis. This observation suggests a coordination between genes involved in the regulation of flavonoids and anthocyanins, considering that anthocyanins are a subclass of water-soluble flavonoids.

#### Sorbitol

Sugar content is one of the most important quality traits perceived by the consumers. The sweetness intensity depends on the overall sugar amount brought by sucrose, glucose, fructose and sorbitol. These first three sugar types were discarded from our analysis as they did not meet the heritability cutoff. Regarding the sorbitol, association signatures were found in chr 1 (~ 27.06 Mbp), chr 2 (~ 3.68 Mbp), chr 6 (~ 28.34 Mbp) and chr 8 (~ 18.84 Mbp). Genetic mapping has been extensively carried out to identify key QTLs responsible for sorbitol biosynthesis. A reliable QTL (qSOR_1) was mapped on the upper region of LG1, nearly 17.5 Mbp upstream of our associated marker (Additional file 2, Figure S10D). Compared to the 9 K association study, no significant association signal was detected on LG1 (Additional file 1, Table S4). On chr 2, we were able to find an adjacent QTL supporting the accuracy of our results [71]. Indeed, qSOR_2 was positioned at ~ 1.2 Mb from our marker SNC_034010.1_3682553.

## Conclusion

The protocol tested here will help other researchers carry out reproducible analyses of ddRAD-seq data and the subsequent variant calling and GWAS model selection. While here it was benchmarked with peach data, it should be equally useful for the study of other crops.

Our study demonstrates the effectiveness of ddRAD-seq genotyping for SNP detection and association studies. Akin to the 9 K SNP array, ddRAD-seq yielded valuable markers strongly supported by stable QTLs. While SNP arrays are designed to target specific genomic regions containing polymorphic loci associated with commercially important traits, ddRAD-seq offers a contrasting approach by randomly sampling the genome without prior knowledge of target regions. This unbiased sampling makes ddRAD-seq particularly well-suited for analyses focused on exploring unexplored biological processes.

In the context of a peach diversity panel, we successfully used our protocol to identify genomic regions and genes influencing major fruit-related traits. The inferred associated SNPs demonstrated reliability, frequently explaining a significant portion of the total phenotypic variance. The survey of candidate genes for these relevant polymorphic sites rendered plenty of genes implicated in various processes. Genes harboring significant markers may be considered as preferential targets for peach breeding. However, given the complexity of the examined traits, future functional validation would provide additional hints to support the breeding efforts.

## Supplementary Information

Supplementary Material 1.

Supplementary Material 2.

## Data Availability

Raw sequence data and final variant call file (vcf) have been deposited in the European Nucleotide Archive (ENA) under the BioProject PRJEB62784 (data will be released upon acceptance of this article). Source code and documentation can be accessed at https://github.com/najlaksouri/GWAS-Workflow.

## Abbreviations

ACNs: Anthocyanins
ddRAD-seq: Double digest restriction-site associated DNA
FF: Flesh firmness
Flv: Contents of flavonoids
FW: Fruit weight
Glu: Glucose
GWAS: Genome wide association analysis
HvD: Harvest date
LD: Linkage disequilibrium LD
MAF: Minor allele frequency
Phen: Total phenolics
RAC: Relative antioxidant capacity
RI: Ripening Index
SNP: Single nucleotide polymorphism
SRB: Sorbitol
SSC: Soluble solids content
SSRs: Simple sequence repeats
Suc: Sucrose
TA: Titratable acidity
TS: Total sugars
