# Supplementary material for: A reproducible ddRAD-seq protocol reveals novel genomic association signatures for fruit-related traits in peach
Source: Plant Methods. 2025 Jul 22;21:101. doi: 10.1186/s13007-025-01415-3 (PMC12285099; doi:10.1186/s13007-025-01415-3)
Supplement: Supplementary file 2 — Supplementary Material 2. [file 13007_2025_1415_MOESM2_ESM.pdf]

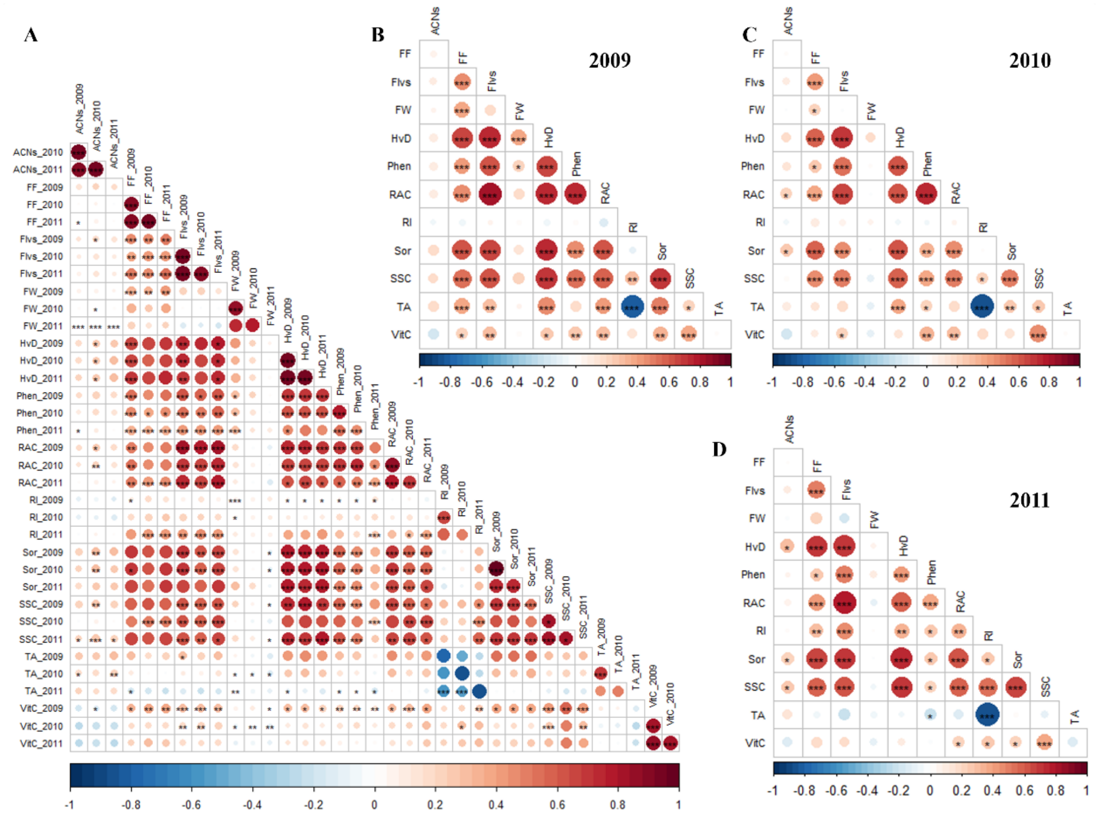

Figure S1. Correlation matrices between phenotypic traits displayed as 4 heatmaps. (**A**) between years (2009, 2010 and 2011), (**B**): 2009, (**C**): 2010, (**D**): 2011. Colors represent positive (red) and negative (blue) correlation. Stars represent statistical significance at a minimum (*P*-value < 0.05*, *P*-value < 0.01** and *P*-value < 0.001***). Harvest date (HvD), fruit weight (FW), flesh firmness (FF), soluble solids content (SSC), titratable acidity (TA), ripening index (RI), content of vitamin C (Vit C), total phenolics (Phen), anthocyanins (ACNs), sucrose (Suc), glucose (Glu), fructose (Fruc), sorbitol (SRB) total sugars (TS) and relative antioxidant capacity (RAC).

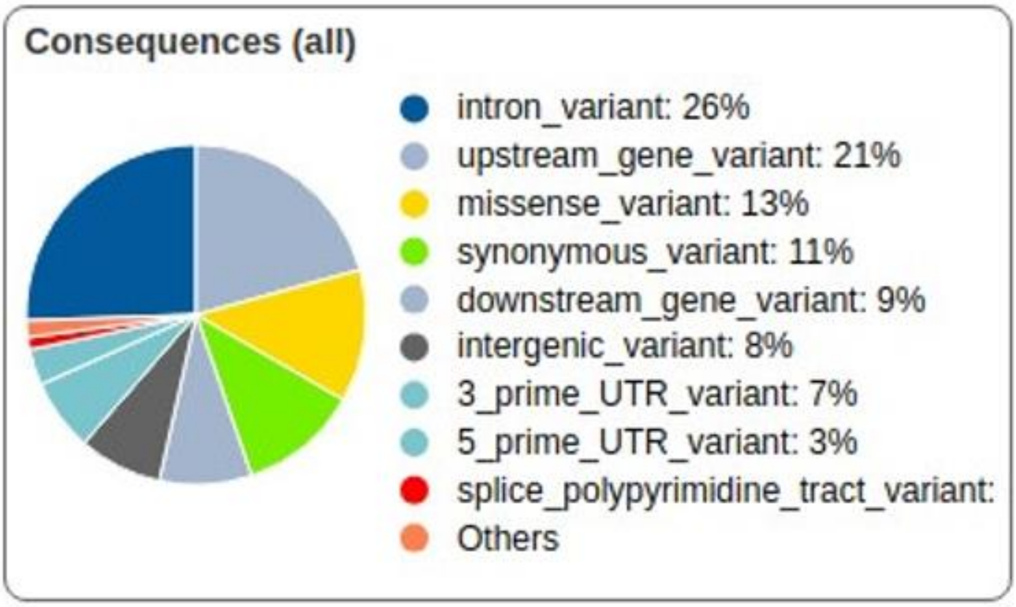

Figure S2. Classification of high-quality SNPs based on their genome location. *Prunus persica* genome assembly (GCA_000346465.2) from Ensembl Plant was used as reference. Missense variant corresponds to a change in the codon resulting in a different amino acid while synonymous variant is defined as codon substitution that does not produce a change the encoded amino acid.

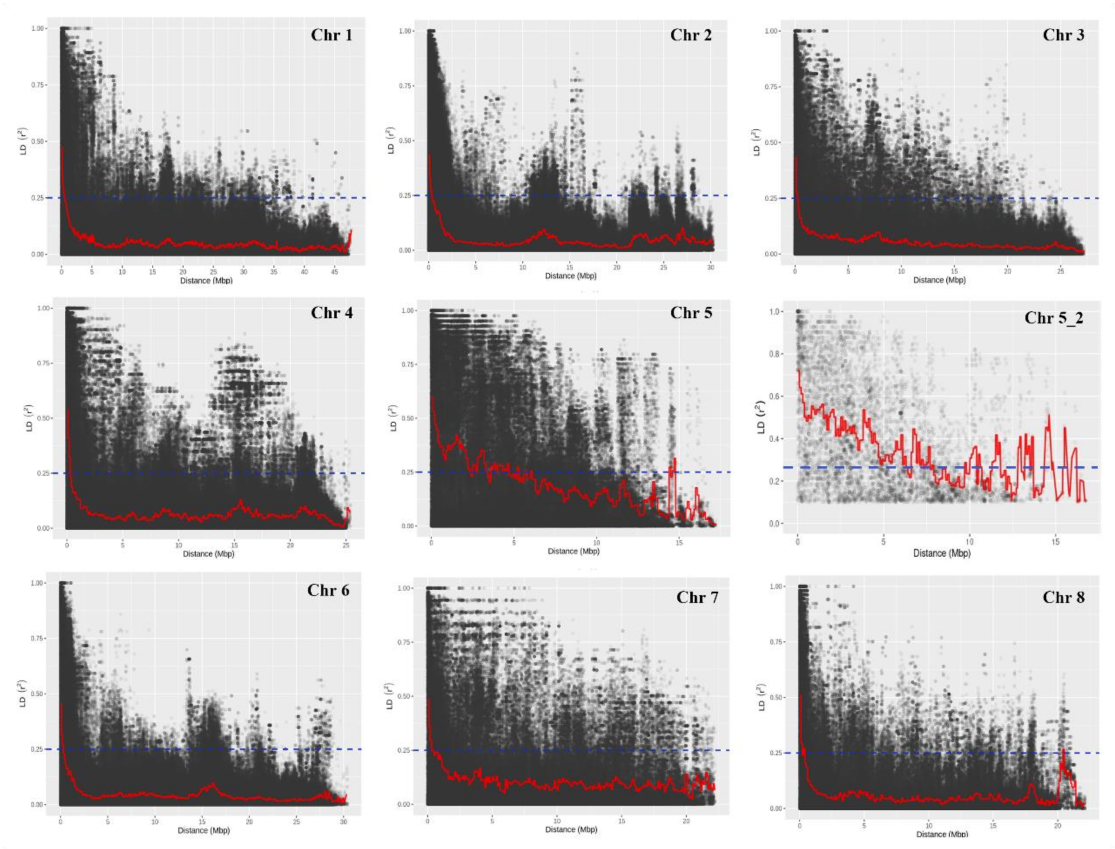

Figure S3. Linkage disequilibrium, measured as r^2, between pairs of polymorphic marker loci against the physical distance (Mbp). Each dot represents the physical distance between each pair of markers along each chromosome's length. Red line represents the trend line estimated as the average of r^2 variation across 1 Mbp bins.

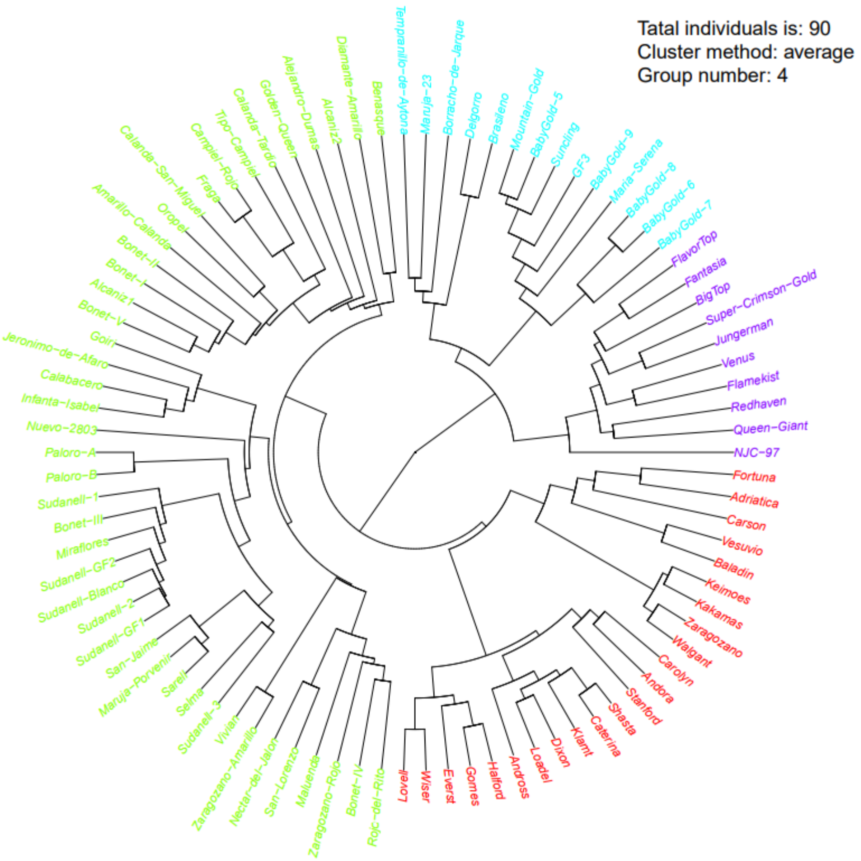

Figure S4. The NJ phylogenetic tree of peach and nectarine cultivars. Accession names within each clade are on the outer ring and are depicted in different colors. Purple color corresponds to clade 1 in the PCA plot (**Figure S3**), blue to clade 2, green to clade 3 and red to clade 4.

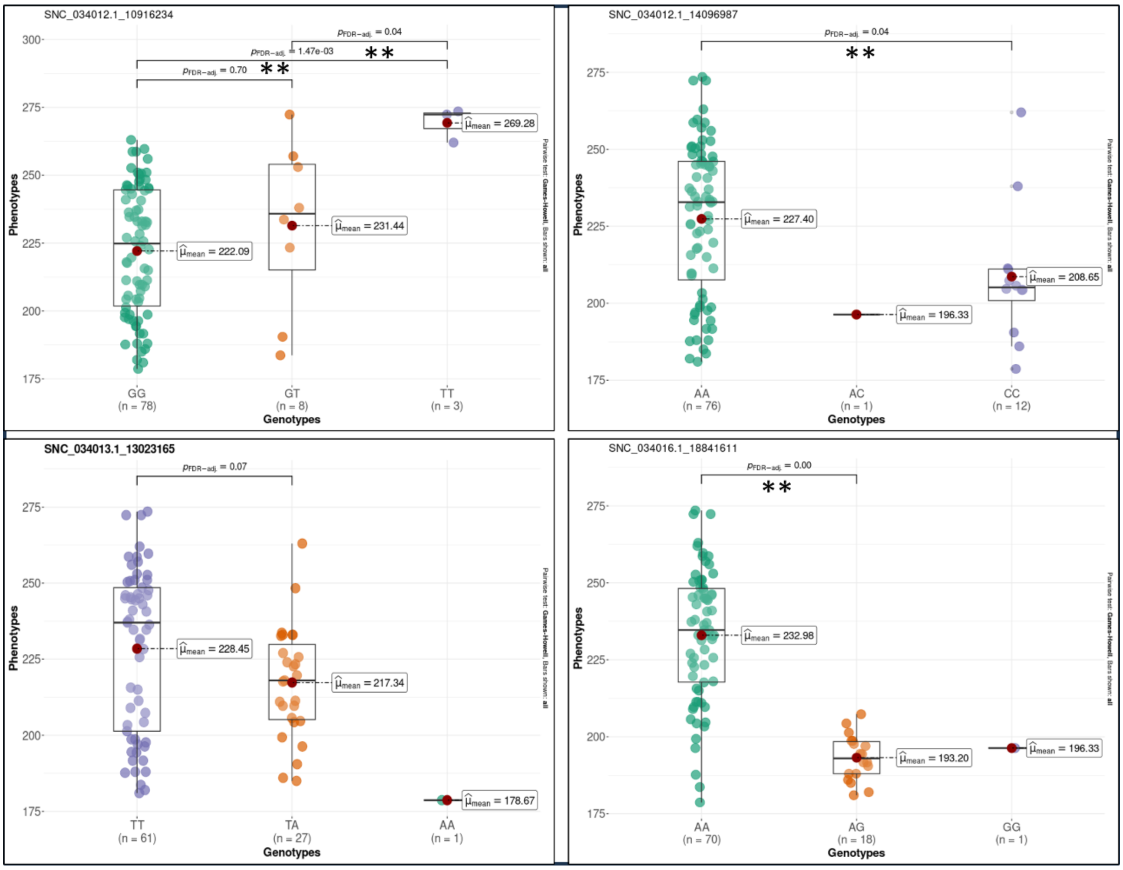

Figure S5. Box plot illustrating allelic effect of significant SNPs on harvest date variation. Y-axis refers to the trait value while x-axis corresponds to the different genotypes (0/0, 0/1 and 1/1). The number of individuals for each genotype is given in parenthesis. Mean values are indicated by red circles and ** indicate significant pairwise comparisons calculated by Games Howel test ($P \leq 0.05$). Lead marker is highlighted in bold.

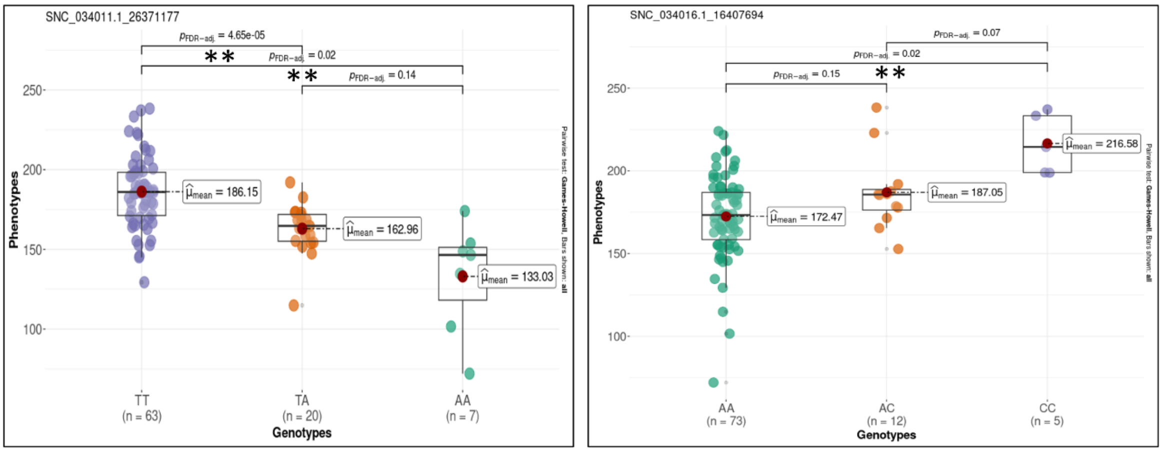

Figure S6. Box plot illustrating allelic effect of significant SNPs on fruit weight. Y-axis refers to the trait value while x-axis corresponds to the different genotypes (0/0, 0/1 and 1/1). The number of individuals for each genotype is given in parenthesis. Mean values are indicated by red circles and ** indicate significant pairwise comparisons calculated by Games Howel test ($P \leq 0.05$).

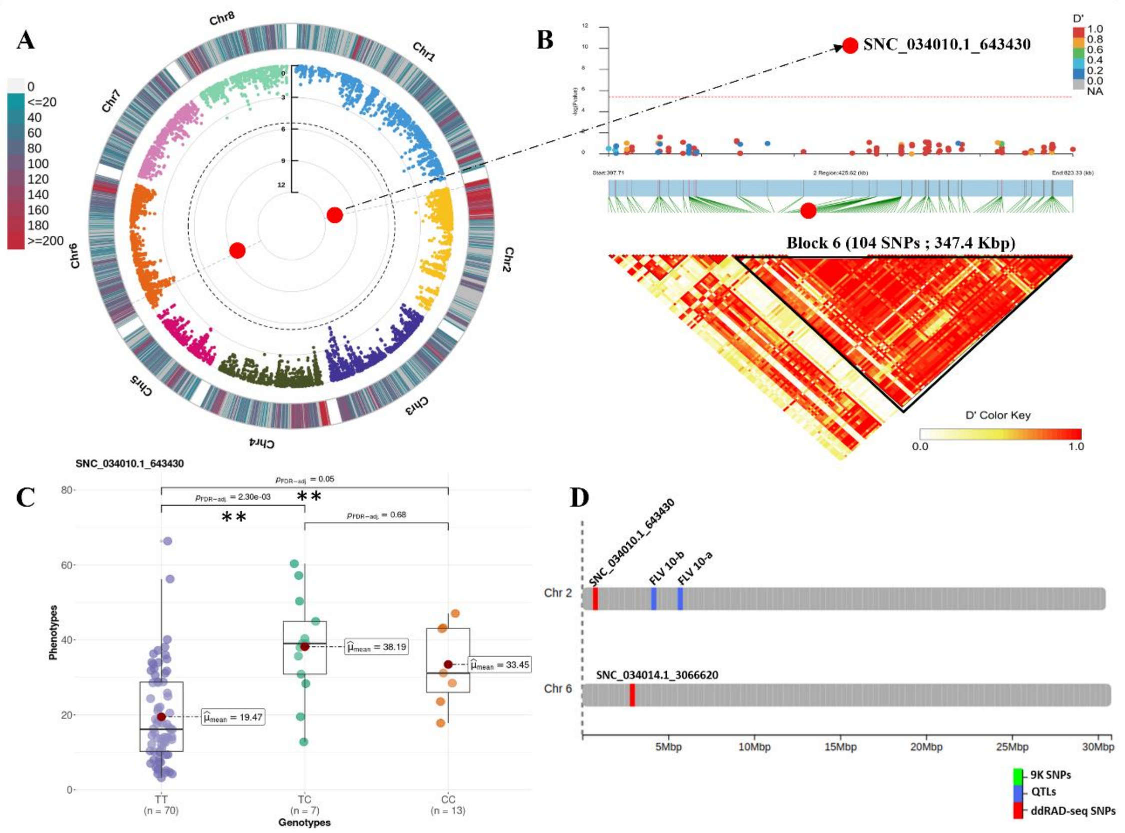

Figure S7. Genome-wide association and LD block analysis for flavonoids content (Flvs). (**A**): Circular Manhattan plot and association signals based on the Blink model. The black dashed circular line corresponds to the Bonferroni adjusted threshold ($-\log_{10}(P)=5.42$). Red and large dots correspond to significantly associated SNPs. Degradation from blue to red indicates the SNP density per 1 Mbp window across peach chromosomes. (**B**): Locus-specific Manhattan plot (upper panel) and LD heatmap (bottom panel) within 250 Kbp on either side of the lead SNP. Pairwise LD measurements are displayed as D' values with a color transition from yellow to red. (**C**): Boxplot depicting the allelic effect of lead SNP on trait variation. The mean value for each genotype is indicated by a red circle, and ** indicates significant pairwise comparisons calculated by the Games–Howel test ($P \leq 0.05$). (**D**): Genomic distribution of significant ddRAD-derived SNPs (red) and reviewed QTLs in the literature (blue).

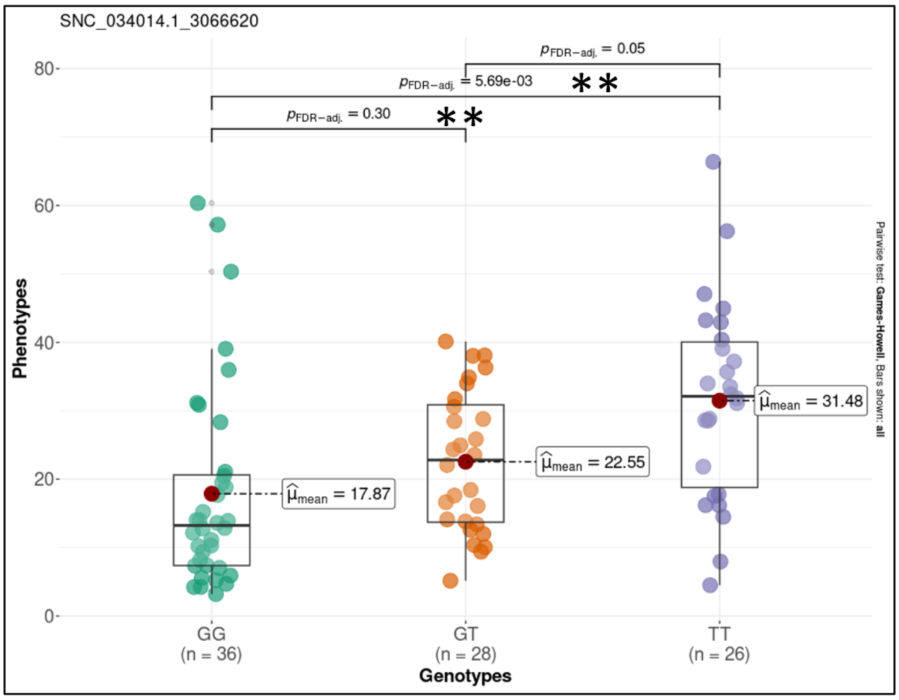

Figure S8. Box plot illustrating allelic effect of significant SNPs on flavonoids. Y-axis refers to the trait value while x-axis corresponds to the different genotypes (0/0, 0/1 and 1/1). The number of individuals for each genotype is given in parenthesis. Mean values are indicated by red circles and ** indicate significant pairwise comparisons calculated by Games Howel test ($P \leq 0.05$).

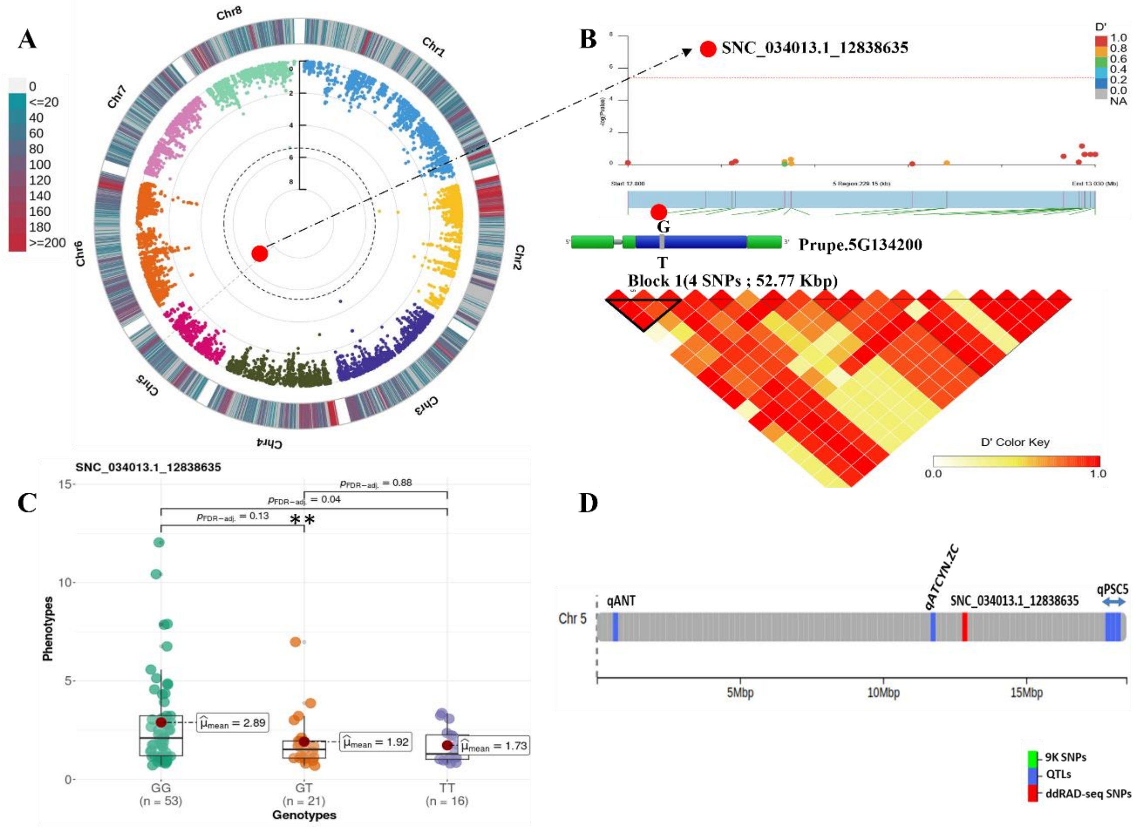

Figure S9. Genome-wide association and LD block analysis for anthocyanins content (ACNs). (**A**): Circular Manhattan plot and association signals based on the Blink model. The black dashed circular line corresponds to the Bonferroni adjusted threshold (-$\log_{10}(P)$=5.42). Red and large dots correspond to significantly associated SNPs. Degradation from blue to red indicates the SNP density per 1 Mbp window across peach chromosomes. (**B**): Locus-specific Manhattan plot (upper panel) and LD heatmap (bottom panel) within 250 Kbp on either side of the lead SNP. Pairwise LD measurements are displayed as D' values with a color transition from yellow to red. (**C**): Boxplot depicting the allelic effect of lead SNP on trait variation. The mean value for each genotype is indicated by a red circle, and ** indicates significant pairwise comparisons calculated by the Games–Howel test ($P \leq 0.05$). (**D**): Genomic distribution of significant ddRAD-derived SNPs (red) and reviewed QTLs in the literature (blue).

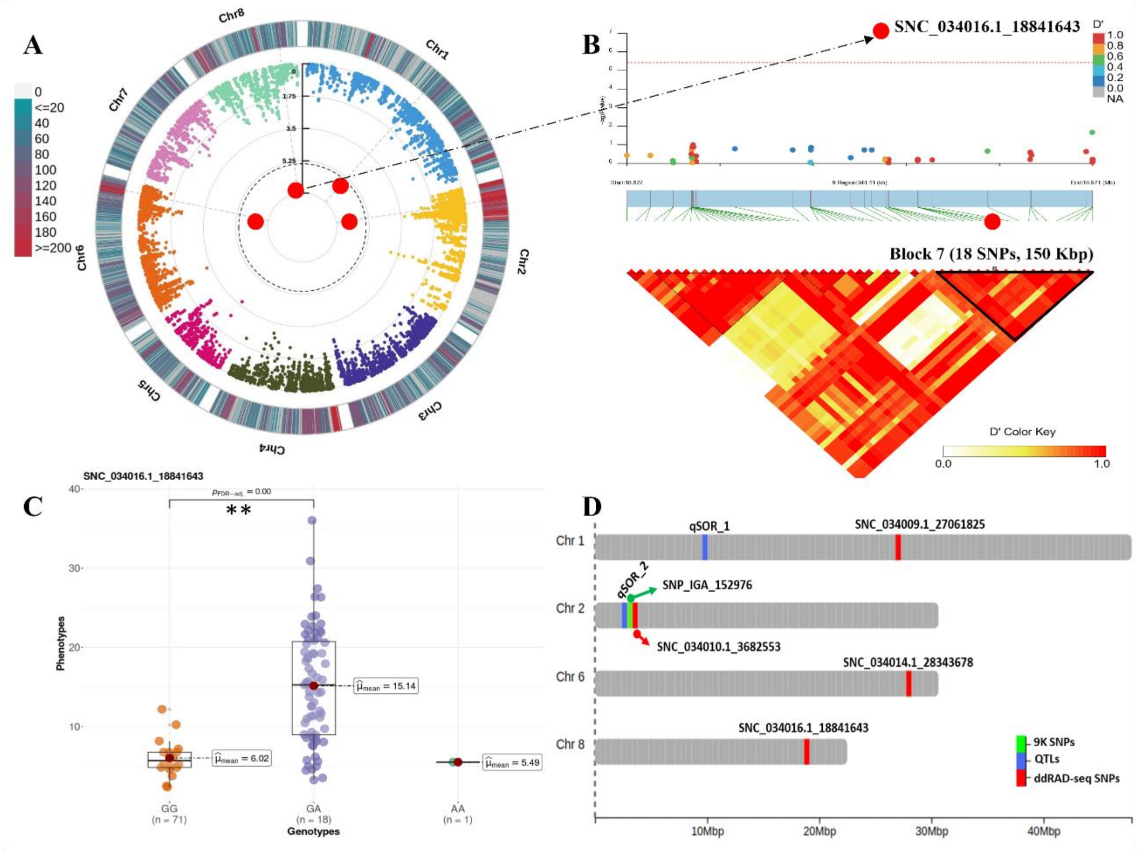

Figure S10. Genome-wide association and LD block analysis for sorbitol content (SRB). (**A**): Circular Manhattan plot and association signals based on the Blink model. The black dashed circular line corresponds to the Bonferroni adjusted threshold ($-\log_{10}(P)=5.42$). Red and large dots correspond to significantly associated SNPs. Degradation from blue to red indicates the SNP density per 1 Mbp window across peach chromosomes. (**B**): Locus-specific Manhattan plot (upper panel) and LD heatmap (bottom panel) within 250 Kbp on either side of the lead SNP. Pairwise LD measurements are displayed as D' values with a color transition from yellow to red. (**C**): Boxplot depicting the allelic effect of lead SNP on trait variation. The mean value for each genotype is indicated by a red circle, and ** indicates significant pairwise comparisons calculated by the Games–Howel test ($P \leq 0.05$). (**D**): Genomic distribution of significant ddRAD-derived SNPs (red) and reviewed QTLs in the literature (blue).

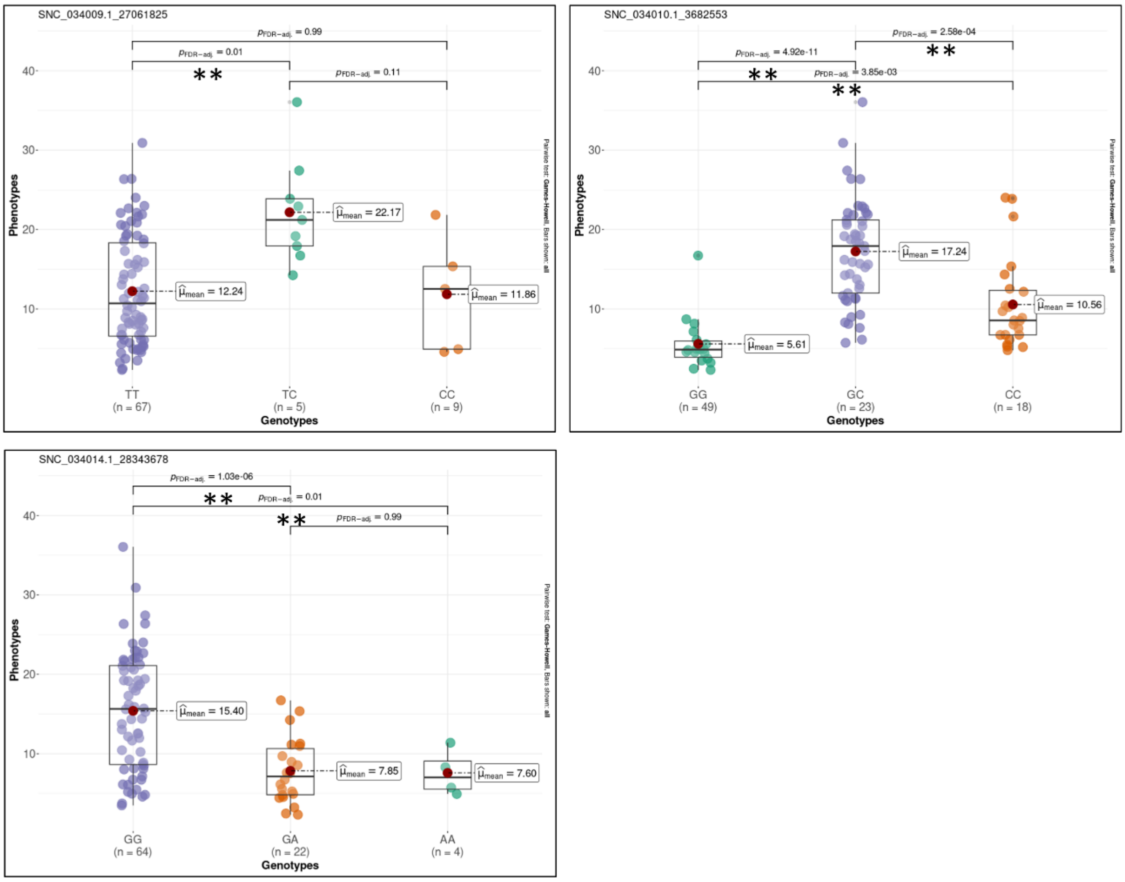

Figure S11. Box plot illustrating allelic effect of significant SNPs on sorbitol content. Y-axis refers to the trait value while x-axis corresponds to the different genotypes (0/0, 0/1 and 1/1). The number of individuals for each genotype is given in parenthesis. Mean values are indicated by red circles and ** indicate significant pairwise comparisons calculated by Games Howel test ($P \leq 0.05$).
